# STUB1 is targeted by the SUMO-interacting motif of EBNA1 to maintain Epstein-Barr Virus latency

**DOI:** 10.1371/journal.ppat.1008447

**Published:** 2020-03-16

**Authors:** Yuyan Wang, Shujuan Du, Caixia Zhu, Chong Wang, Nuoya Yu, Ziqi Lin, Jin Gan, Yi Guo, Xinxin Huang, Yuping He, Erle Robertson, Di Qu, Fang Wei, Qiliang Cai

**Affiliations:** 1 MOE&NHC&CAMS Key Laboratory of Medical Molecular Virology, Department of Medical Microbiology and Parasitology, Biosafety Level 3 Laboratory, School of Basic Medical Science, Shanghai Medical College, Fudan University, Shanghai, P. R. China; 2 ShengYushou Center of Cell Biology and Immunology, School of Life Science and Biotechnology, Shanghai Jiao Tong University, Shanghai, P. R. China; 3 Department of Gynecology, Key laboratory of AIDS immunology of Ministry of Health, First Affiliated Hospital of China Medical University, Shengyang, P. R. China; 4 Technical Center For Animal, Plant and Food Inspection and Quarantine of Shanghai Customs, Shanghai, P. R. China; 5 Department of Otorhinolaryngology-Head and Neck surgery, Department of Microbiology, Perelman School of Medicine, University of Pennsylvania, Philadelphia, Pennsylvania, United States of America; 6 Expert Workstation, Baoji Central Hospital, Baoji, P. R. China; National Cancer Institute, UNITED STATES

## Abstract

Latent Epstein-Barr virus (EBV) infection is strongly associated with several malignancies, including B-cell lymphomas and epithelial tumors. EBNA1 is a key antigen expressed in all EBV-associated tumors during latency that is required for maintenance of the EBV episome DNA and the regulation of viral gene transcription. However, the mechanism utilized by EBV to maintain latent infection at the levels of posttranslational regulation remains largely unclear. Here, we report that EBNA1 contains two SUMO-interacting motifs (SIM2 and SIM3), and mutation of SIM2, but not SIM3, dramatically disrupts the EBNA1 dimerization, while SIM3 contributes to the polySUMO2 modification of EBNA1 at lysine 477 *in vitro*. Proteomic and immunoprecipitation analyses further reveal that the SIM3 motif is required for the EBNA1-mediated inhibitory effects on SUMO2-modified STUB1, SUMO2-mediated degradation of USP7, and SUMO1-modified KAP1. Deletion of the EBNA^SIM^ motif leads to functional loss of both EBNA1-mediated viral episome maintenance and lytic gene silencing. Importantly, hypoxic stress induces the SUMO2 modification of EBNA1, and in turn the dissociation of EBNA1 with STUB1, KAP1 and USP7 to increase the SUMO1 modification of both STUB1 and KAP1 for reactivation of lytic replication. Therefore, the EBNA1^SIM^ motif plays an essential role in EBV latency and is a potential therapeutic target against EBV-associated cancers.

## Author summary

The Small Ubiquitin-related modifier (SUMO) modification of proteins is a reversible post-translational regulation involved in control of gene transcription, among other functions. Epstein-Barr virus (EBV) infects most people worldwide and contributes to the development of several types of cancers due to its ability to induce cell proliferation and survival. EBNA1 is expressed in all forms of EBV-associated tumors. In this study, we found that EBNA1 contains a SUMO-interacting motif (SIM) named EBNA1^SIM^, which is required for EBNA1 to exert inhibitory effects on a SUMO2-modified complex (SC2) including STUB1, KAP1 and USP7. Disruption of EBNA1^SIM^ leads to loss of both EBNA1-mediated viral episome maintenance and lytic gene silencing. Importantly, hypoxia-mediated reactivation of viral lytic replication induces the EBNA1 dissociation from STUB1 in the SC2 complex. This discovery not only opens a new insight on the interplay between host and virus, but it also provides a therapeutic target specific against EBV-associated cancers.

## Introduction

Epstein-Barr virus (EBV) was the first human tumor virus to be discovered. It was identified from Burkitt’s lymphoma (BL) in 1964, and accounts for approximately 2% of all cancer deaths in the world to date [1]. EBV belongs to the gammaherpesvirus and has a typical herpesvirus life cycle: latency and lytic replication. Over 90% of the worldwide population carries the latent EBV with an asymptomatic life-long infection [2]. Under certain conditions that are still not well known, the virus can cause human cancers including lymphoid and epithelial malignancies [3,4]. The EBV-encoded nuclear antigen 1 (EBNA1) is the only viral protein consistently expressed in all EBV-associated malignancies [5]. EBNA1 is essential for viral genome DNA replication and maintenance and as well as controlling viral gene expression [2]. It has been demonstrated that EBNA1 is a multiple functional protein that interacts with numerous host proteins, such as EBP2 [6], USP7 [7], casein kinase 2 [8], Tankyrase 1 [9], PRMT5 and P32/TAP [10], among others, playing a critical role in the onset, progression, and/or maintenance of its associated tumors [10,11].

During the proliferation of EBV-infected cells, the viral DNA replication and episome maintenance is dependent on the interaction of EBNA1-bound viral episome with chromatin [11]. The ability of EBNA1 to bind to the origin of viral replication (*OriP*) has been demonstrated to be critical for driving the latent cycle of viral episome DNA replication and segregation, and to maintain its stability in proliferating cells [2]. *OriP* is comprised of two functional elements, the dyad symmetry (DS) element and the family of repeats (FR) [12]. To initiate the viral latent DNA replication, a dimer-dimer EBNA1 interaction is required to bind with four recognition sites within the DS sequence. This cooperativity highly relies on the DNA binding and dimerization domain (DBD/DD) of EBNA1, which is located between the amino acids (aa) 459 and 607 [13,14]. In contrast, the binding of FS (a cluster of 20 tandem 30-bp repeats) with EBNA1 is important for governing the mitotic segregation of the EBV genomes and maintaining the stability of the episome in the EBV latently infected cells [15,16]. In addition, the complex of EBNA1 bound to FR can also act as a transcriptional enhancer to activate the expression of other EBV latent genes [15,17,18]. More importantly, the viral episome is tightly dependent on EBNA1 to be tethered with host chromosomes during the mitotic process [16]. The amino-terminal region including arginine-glycine (G/R) repeats (aa 33 to 53) of EBNA1 is involved in the chromosome binding directly or indirectly through interaction with cellular proteins, such as hEBP2 and RCC1 [6,19–23].

Emerging studies have shown that EBNA1 can interact with many host proteins to exert different functions, including enhancing transcription of viral genes, regulating many host signaling pathways in the different cell types, and viral latency [2]. For example, it has been found that EBNA1 interacts with USP7 and casein kinase 2 (CK2) to trigger PML ubiquitylation and degradation [24,25]. To promote the survival of EBV latently-infected cells with DNA damage, EBNA1 blocks the p53-USP7 interaction, which results in malignant transformation [7,24,26]. In addition, it has also been demonstrated that the central glycine-alanine repeat (GAr) of EBNA1 plays a critical role in the immune evasion, through suppression of the translation of its own mRNA in a cis-regulated mode [27]. The nucleolin, a DNA/RNA binding protein, can directly interact with the G-quadruplexes of the GAr-encoding mRNA sequence to enhance GAr-based inhibition of EBNA1 protein expression, and in turn relieve the suppression of both its expression and antigen presentation [28].

Small ubiquitin-related modifier (SUMO) modification of proteins is a reversible post-translational modification that plays a critical role in the regulation of cellular and viral gene transcription, as well in response to hypoxic stress [29–31]. So far, three major SUMO isoforms (SUMO1, SUMO2 and SUMO3) have been identified in mammalian cells. SUMO1 is the major SUMO in human cells; SUMO2 and SUMO3 are highly homologous (often referred to as SUMO2/3), having 50% identical in sequence to SUMO1 [32]. Like ubiquitylation, SUMO conjugation occurs through an enzyme cascade: E1 activating enzyme (Uba2-Aos1), E2 conjugating enzyme (Ubc9), and E3 ligase [33]. While distinct from ubiquitylation, SUMO conjugation to a target substrate often requires a consensus sequence ΨKxE/D (Ψ, large hydrophobic residue; x, any amino acid) around the target lysine [34]. Protein modification by SUMO can affect the regulation of diverse cellular processes, including signal transduction, protein trafficking, chromosome segregation, and DNA repair [35]. In addition to its conjugation to substrates through a covalent ligation, it has been demonstrated that SUMO can also non-covalently bind to other proteins that have a consensus SUMO-interacting motif (SIM) [36–38]. Thus, the biological functions of the SUMO-modified substrates rely on their ability to interact with other effector containing SIM motifs. Increasing studies have suggested that the SUMO conjugation pathway may play an important role in several aspects of herpes viral replication and pathogenesis, such as the maintenance and stability of the viral episome DNA during latency, the regulation of several herpesviral proteins that disrupt the disassembly of PML-nuclear body, and the attenuation of the host interferon responses. For instance, our previous studies have shown that LANA, which is encoded by Kaposi’s sarcoma-associated herpesvirus (KSHV), is involved in the recruitment of various SUMO-modified protein, including KAP1 (a regulator for chromatin remodeling), through a unique SUMO-interacting motif to maintain the DNA passage of viral episome during latency [29]. Furthermore, the regulation of several herpesviral proteins that disrupts the disassembly of the PML-nuclear body ensures the herpesviral lytic replication in a SUMO-dependent manner; examples of these proteins include BZLF1 and BGLF4 of EBV [39,40], KSHV encoded K-Rta [41], K-vZIP and vIRF-3 [42,43], ICP0 of HSV1 [44], and IE1 protein of human cytomegalovirus (HCMV) [45]. Finally, to attenuate the host’s interferon responses, K-bZIP has been found to inhibit interferon-α (IFN-α) signaling in a SUMOylation-dependent manner [35,46], the EBV latent membrane protein 1 (LMP1) induces IRF-7 SUMOylation to block the host immune response [47], and vesicular stomatitis virus (VSV) infection can trigger SUMOylation of both IRF-3 and IRF-7 to attenuate the interferon production [48]. However, whether EBNA1, as a homolog of LANA, exerts its functions through regulation of SUMO signaling pathways remains largely unclear.

In this study, we demonstrate that EBNA1 contains a SIM (EBNA1^SIM^) that is required for the EBNA1-mediated inhibitory effects on a SUMO2-modified complex including STUB1, KAP1 and USP7. Functional analysis revealed that the EBNA1^SIM^ is essential for EBNA1-mediated DNA binding and maintenance of the EBV episome. Moreover, hypoxic stress induces SUMO2 modification of EBNA1, and in turn the dissociation of EBNA1 with STUB1, KAP1, and USP7 to increase the SUMO1 modification of both KAP1 and STUB1 for reactivation of lytic replication. This provides a potential therapeutic specific target against EBV-associated cancers.

## Results

### EBNA1 contains two SUMO-interacting motifs

**Fig 1:**
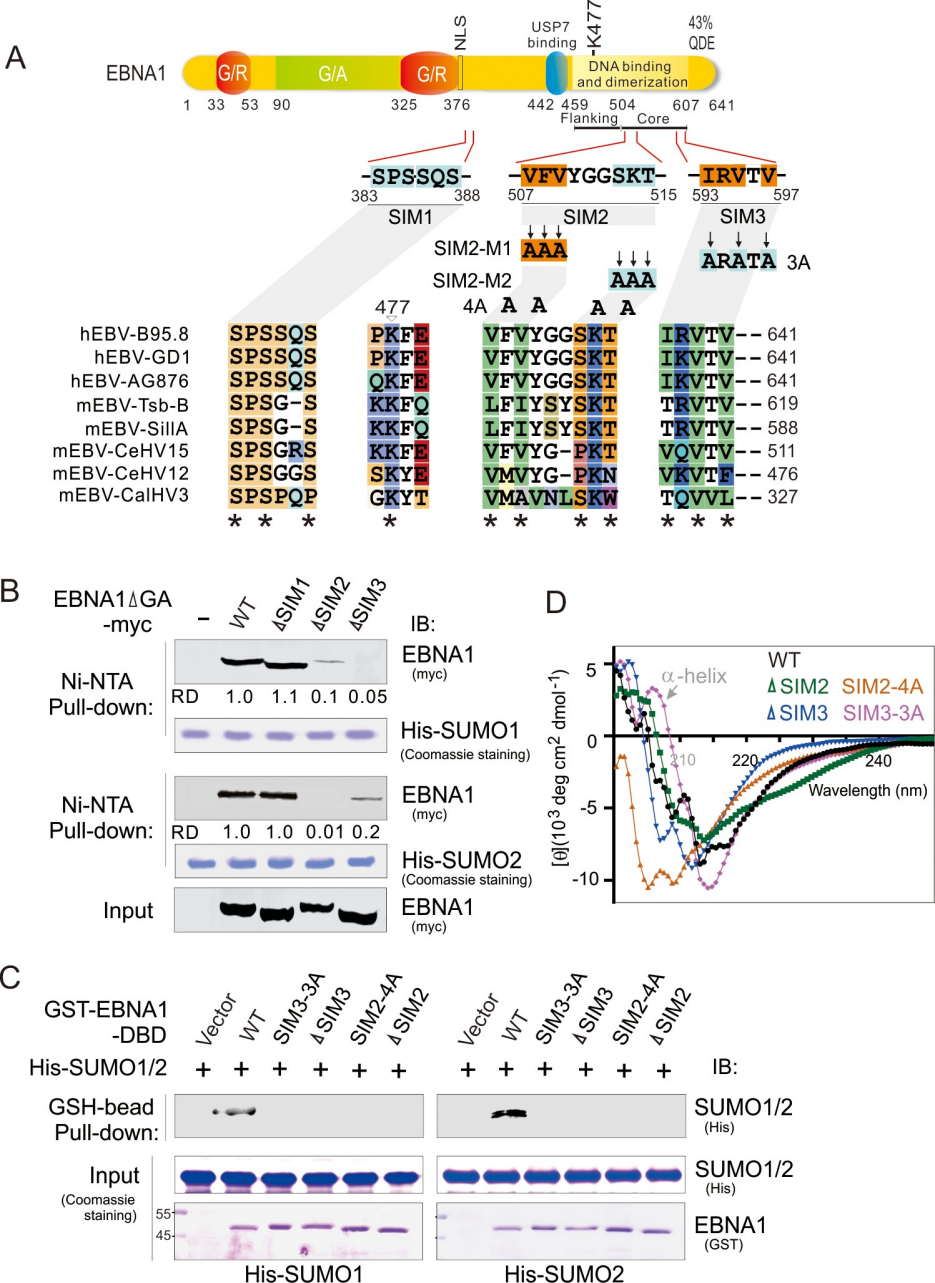
EBNA1 contains two SUMO-interacting motifs. (**A**) Schematic representation of different EBNA1 mutants with mutation of SUMO-interacting motifs (SIMs). The residue positions of each putative SIM1, SIM2, and SIM3 of EBNA1, site-mutation of four SIM mutants (SIM2-m1, SIM2-m2, SIM2-4A, and SIM3-3A), and G/A and G/R rich region are indicated. K477 is the putative SUMOylation site based on SUMOplotanalysis. The amino acid alignment of SIM regions of EBNA1 homologs encoded by different EBV strains of human and other primate is shown at the bottom panel. The asterisk indicates highly conserved residues. QDE, acidic domain. NLS, nuclear localization sequence. (**B**) The SIM2 and SIM3 of EBNA1 are required for interaction with SUMO1/2. HEK293T cells were individually transfected with expression plasmids as indicated in the figure. At 48 h post-transfection, whole cell extracts (Input) were subjected to a pulldown with His-SUMO1 or His-SUMO2 recombinant proteins with Nickel-agarose (Ni-NTA) beads, followed by immunoblotting (IB) with anti-myc antibodies. His-SUMO1 and His-SUMO2 recombinant proteins are shown by Coomassie staining. The relative density (RD) of EBNA1-interacting SUMO1 or SUMO2 is quantified and shown on the right panel. Input was used at 5%. (**C**) The SIM2 and SIM3 motifs of EBNA1 are required to directly bind with SUMO1/2 *in vitro*. Equal protein amounts of purified His-SUMO1/2 (Input) were individually incubated with wild type (WT) EBNA1 and its SIM2/3 mutants of GST-fusion proteins purified from *E*.*coli* expression system and pulled down with Glutathione (GSH)-sepharose beads. Bound complexes were analyzed by immunoblotting (IB) with the indicated antibodies. The recombinant proteins are shown by Coomassie staining at the bottom panel. (**D**) Circular dichroism (CD) spectra for wild type EBNA1 and its SIM2/3 mutants in phosphate buffer (pH = 7.2).

To investigate whether EBNA1 is involved in the regulation of SUMO signaling pathway, we performed co-immunoprecipitation (co-IP) assays by co-expressing myc-tagged EBNA1 with FLAG-tagged SUMO1 or SUMO2 in HEK293T cells. The results showed that EBNA1 was associated with both SUMO1 and the SUMO2 modified substrate [(SUMO1/2)n-sb] with high molecular weight (>170kDa) to some extent, although we found a moderately higher association of EBNA1 with (SUMO1)n-sb than with (SUMO2)n-sb (S1 Fig, top panels, compare lane 4 with lane 8). To further identify whether EBNA1 directly interacts with the SUMO1/2 molecule, we first aligned the amino acid sequence of EBNA1 with the conserved sequence of a cellular SIM as described previously [29,37,38]. As shown in Fig 1A, the results identified three potential SIM-like motifs, SIM1, SIM2, and SIM3, which are located within the residues 383 to 388, 507 to 515, and 593 to 597 of EBNA1, respectively. Intriguingly, these three SIM-like motifs are highly conserved in the homologs of EBNA1 encoded by different human EBV strains including B95.8, GD1 and AG876, as well as five monkey EBV strains (Tsb-B, SillA, CeHV15, CeHV12, and CalHV3) based on the sequence analysis (Fig 1A, bottom panels).

To prove that these SIM motifs are required for EBNA1 to interact with the SUMOs, we generated a series of EBNA1 mutants with the deletion of different SIM motifs (Fig 1A), followed by *in vitro* pull-down assays with His-tagged SUMO1 or SUMO2 recombinant proteins. The results showed that deletion of either the SIM2 or SIM3 motif strikingly reduced the binding ability of EBNA1 to His-SUMO1 or His-SUMO2 recombinant protein, while deletion of SIM1 presented no effect (Fig 1B). The same results were obtained from His-SUMO1/2 recombinant protein was reverse pulled down *in vitro* by using Glutathione-sepharose beads targeting GST-fusion wild type EBNA1 DBD protein instead of its mutants (ΔSIM2, SIM2-4A, ΔSIM3 or SIM3-3A) purified from the *E*.*coli* expression system (Fig 1C), further confirming that the SIM motif of EBNA1 could directly interact with the SUMO molecule. To address whether the mutation of EBNA1^SIM^ impairs the folding of the EBNA1 DBD, we have also performed CD analysis of purified proteins of wild type EBNA1 and its SIM2/3 mutants with deletion or site mutation, and found that both deletion of SIM2/3 and site mutation of SIM3 mutants did not significantly impair the α-helix folding structure of EBNA1 DBD, while the mutation of SIM2-4A dramatically disrupts the formation of α helix at the wavelength between 200 and 210 nm (Fig 1D). To further elucidate the role of both SIM2 and SIM3 motifs, we also determined the interaction of EBNA1 with HA-tagged exogenous Ubc9 (E2 conjugation enzyme) by co-expression in HEK293 cells, followed by co-immunoprecipitation and immunoblotting assays. The results showed that deletion of either SIM2 or SIM3 did efficiently reduce the binding of EBNA1 with SUMO-modified Ubc9 (Ubc9-SUMO), albeit no significant effect on the ability of EBNA1 binding with Ubc9 when GA is deleted (supplementary S2 Fig). This suggests that the EBNA1^SIM^ motif is required to interact with SUMO molecules.

### Deletion of SIM2, not SIM3, impairs EBNA1 dimerization, and SIM3 contributes to poly-SUMO2 modification of EBNA1 *in vitro*

**Fig 2:**
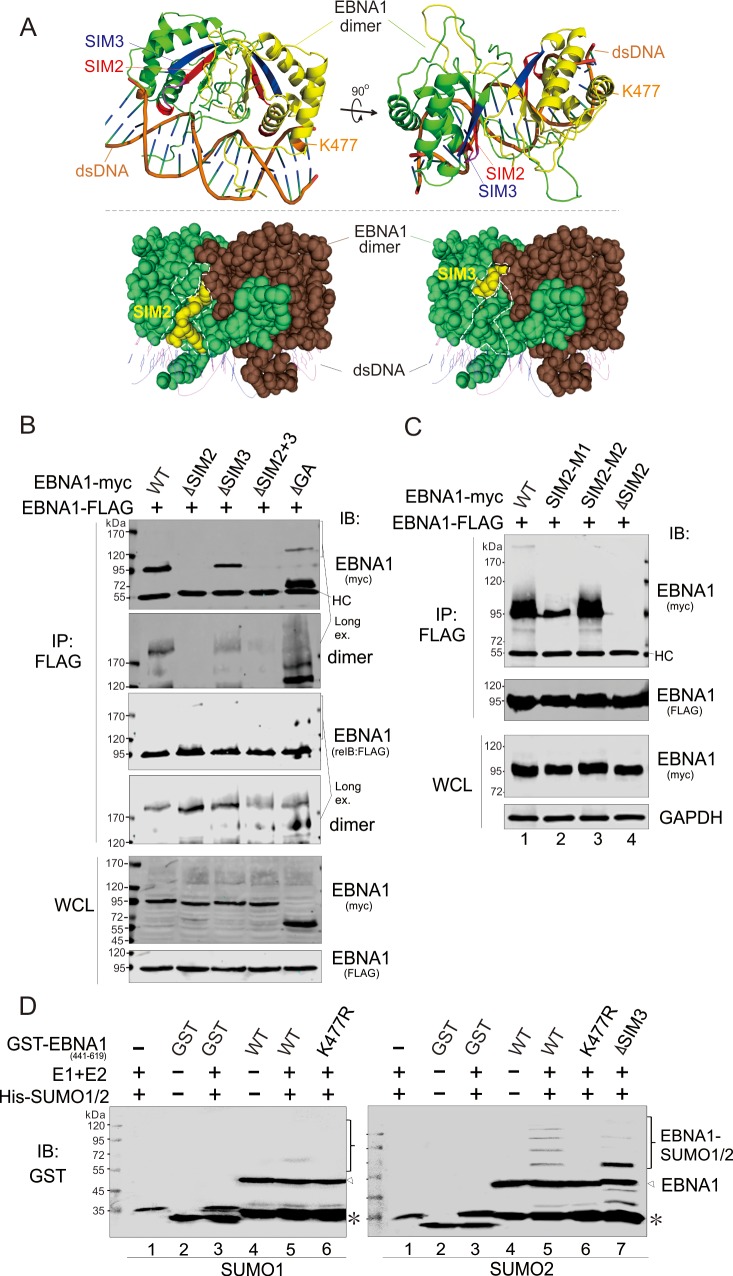
The EBNA1^SIM^ deletion impairs EBNA1 dimerization and SUMOylation. (**A**) Illustration of the position of SIM2, SIM3 and K477 in the 3D structure of EBNA1 (PDB#1B3T, from http://www.rcsb.org/pdb/home/home.do) predicted by Robetta server (http://robetta.bakerlab.org/). Double-stranded DNA (dsDNA, brown), lysine 477 (K477, orange) and the SIM2 (red) and SIM3 (blue) motifs within EBNA1 dimer (green and yellow) are highlighted. *Bottom panel*, the SIM2 and SIM3 motifs of EBNA1 are marked in yellow. (**B**) The deletion of SIM2 but not SIM3 dramatically abolishes dimer formation of EBNA1. HEK293T cells were co-transfected with expression plasmids as indicated. WCL were harvested at 48 h post-transfection, and individually subjected to co-IP and IB as indicated. HC, heavy chain. (**C**) The intact SIM2 motif is required for EBNA1 dimer formation. HEK293T cells co-transfected with expression plasmids as indicated, were subjected to the co-IP and IB analysis as described in panel B. (**D**) Deletion of the SIM3 motif, but not its single K477R mutation, facilitates mono-SUMOylation of EBNA1 *in vitro*. Purified proteins of GST-fusion WT EBNA1 (441–619) and its mutants (K477R, ΔSIM3) were individually incubated with the indicated recombinant proteins (SUMOylation enzymes E1, E2, and SUMO1/2 with His tag). Reaction mixtures were analyzed by SDS-PAGE, followed by IB with a GST antibody. The asterisk indicates an uncharacterized crossing-reaction band.

Since both SIM2 and SIM3 motifs are located within the DNA binding and dimerization domain of EBNA1, to elucidate the role of the two SIM motifs on EBNA1 functions, we first analyzed the location of SIM2 and SIM3 motifs on the 3D structure of EBNA1 protein (PDB ID: 1B3T, by software pymol-v1.3r1-edu-Win32)[13,49]. Interestingly, we found that both SIM2 and SIM3 motifs locate side-by-side at the surface of the EBNA1 protein, and both of them are also very close to the junction edge of the EBNA1 dimer (Fig 2A). This suggests that these two SIM motifs may be involved in the dimer formation of EBNA1 and its related biological functions.

To determine whether the SIM2 or SIM3 motif impairs the EBNA1 dimerization, we performed a co-IP assay to detect the interaction of FLAG-tagged EBNA1 with myc-tagged wild type (WT) EBNA1 or its SIM-deleted mutants in HEK293 cells. As shown in Fig 2B, deletion of SIM2, but not that of the SIM3 motif, completely disrupted the dimer formation of EBNA1, when compared with WT EBNA1 or its GA-deleted mutant. In the view of the fact that the SIM2 motif is composed of 9 aa residues with two potential sub-SIM motifs (VxV and SxT), we also generated another two mutants of EBNA1 with point mutation on either one of the two sub-SIM motifs (SIM2-M1 and SIM2-M2) (Fig 1A), and performed similar co-immunoprecipitation assays. The results showed that, unlike the M2 mutation of SIM2, the M1 mutation could more efficiently inhibit the formation of EBNA1 dimer, albeit the inhibition efficiency of M1 was much lower than that in the SIM2-deleted mutant (Fig 2C, compare lanes 2, 3 with 4), which confirmed that deletion of SIM2, but not SIM3, impairs EBNA1 dimer formation.

In addition, we also noticed that lysine 477 (K477) within the DNA-binding domain of EBNA1 is a putative SUMOylated site with an associated highly probability (> 0.90) based on the prediction analysis (Fig 1A). To determine whether K477 is the SUMOylated site of EBNA1 and whether SIM3 contributes to its SUMOylation, we individually generated and purified the GST-tagged recombinant protein of the DNA-binding truncated mutant of EBNA1 (GST-EBNA1_441-619_) and its K477R or SIM3-deleted mutants using an *E*.*coli* expression system, and then carried out *in vitro* SUMOylation assays by incubation with His-tagged SUMOylation E1 and E2 enzymes in the presence of SUMO1 or SUMO2. Strikingly, the results showed that the K477R mutation could efficiently reduce both the SUMO1 and SUMO2 modification of EBNA1 *in vitro* (Fig 2D, compare lanes 6 and 5). In contrast, the deletion of the SIM3 motif remarkably reduced the poly-SUMO2 modification of EBNA1 (Fig 2D, compare lanes 7 and 5), indicating that the SIM3 motif contributes to the poly-SUMO2 modification of EBNA1, and the loss of poly-SUMO2 chain in the absence of SIM3 motif could be due to the lower affinity of EBNA1 dimer association with SUMO2.

**Fig 3:**
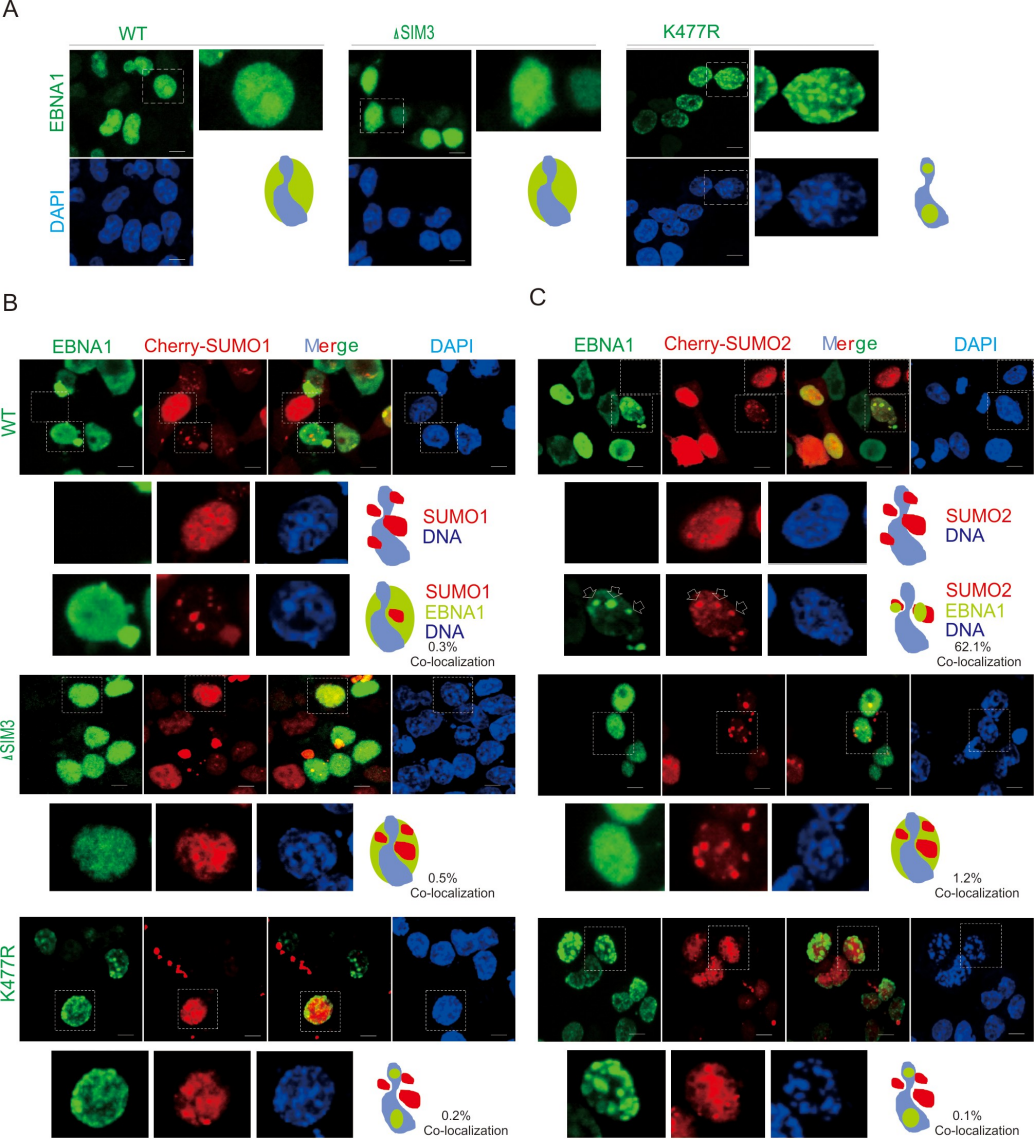
Deletion of EBNA1^SIM^ reduces the co-localization of EBNA1 with SUMO2. (**A**) K477R mutation but not SIM3 deletion results in punctate localization of EBNA1 on chromatin DNA. HEK293 cells transfected WT, SIM3-deleted (ΔSIM3) or the K477R mutant EBNA1 with myc tag were individually cultured on coverslips, fixed with 3% paraformaldehyde, and then stained with anti-myc antibody as indicated. Nuclei were counterstained with DAPI. Scale bars, 5 μm. Right panels show the magnified view and schematic of localization of EBNA1 (green) with host chromatin DNA (blue). (**B**) WT EBNA1, but not its SIM-deleted or K477R mutant, reduces the formation of SUMO1 punctate dots. HEK293 cells co-transfected Cherry-SUMO1 with vector alone, WT or SIM-deleted (ΔSIM3) or the K477R EBNA1 mutant with myc tag vectors, were individually subjected to immunofluorescence assays as described in *panel A*. The magnified view and schematic of localization of EBNA1 (green), SUMO1 (red) and host chromatin DNA (blue) and the percentage of co-localization from 30 counted cells are shown in the bottom panels. (**C**) WT EBNA1, but not its SIM-deleted or K477R mutant, co-localizes with SUMO2 as punctate dots. HEK293 cells co-transfected Cherry-SUMO2 with vector alone, WT or SIM-deleted (ΔSIM3) or the K477R EBNA1 mutant with myc tag vectors, was individually subjected to immunofluorescence assays as described in panel A. The magnified view and schematic of localization of EBNA1 (green), SUMO2 (red) and host chromatin DNA (blue) and the percentage of co-localization from 30 counted cells are shown in the bottom panels. The co-localization of WT (not K477R) EBNA1 with SUMO2 was highlighted by arrows.

To further confirm that both the K477 residue and the SIM3 motif are required for SUMOylation of EBNA1, we investigated the subcellular localization of both EBNA1 and SUMO1 (or SUMO2) by co-expressing cherry-labeled SUMO1/2 with the WT EBNA1 and its K477R or SIM3-deleted versions in HEK293 cells. Intriguingly, in the absence of exogenous SUMO1/2 molecules, the results showed that both WT EBNA1 and its SIM3-deleted mutant are diffusely distributed within the nuclear compartment, while the K477R mutation resulted in the clear co-localization of EBNA1 with DNA as punctate dots (Fig 3A). In contrast, in the presence of exogenous SUMO1 or SUMO2, although EBNA1 could reduce the number of both SUMO1 and SUMO2 of punctate dots to some extent, only SUMO2 but not SUMO1 co-localization with EBNA1 as the punctate dots appeared (Fig 3B, top panel), which could be efficiently disrupted by mutation of K477R or the SIM3-motif deletion (Fig 3B, middle and bottom panels). This consistently supports the notion that SUMOylation of EBNA1 relies on both Lysine 477 and the SIM3 motif.

### The EBNA1^SIM^ motif is essential for EBV episome maintenance, silencing ZTA expression and blocking lytic replication

**Fig 4:**
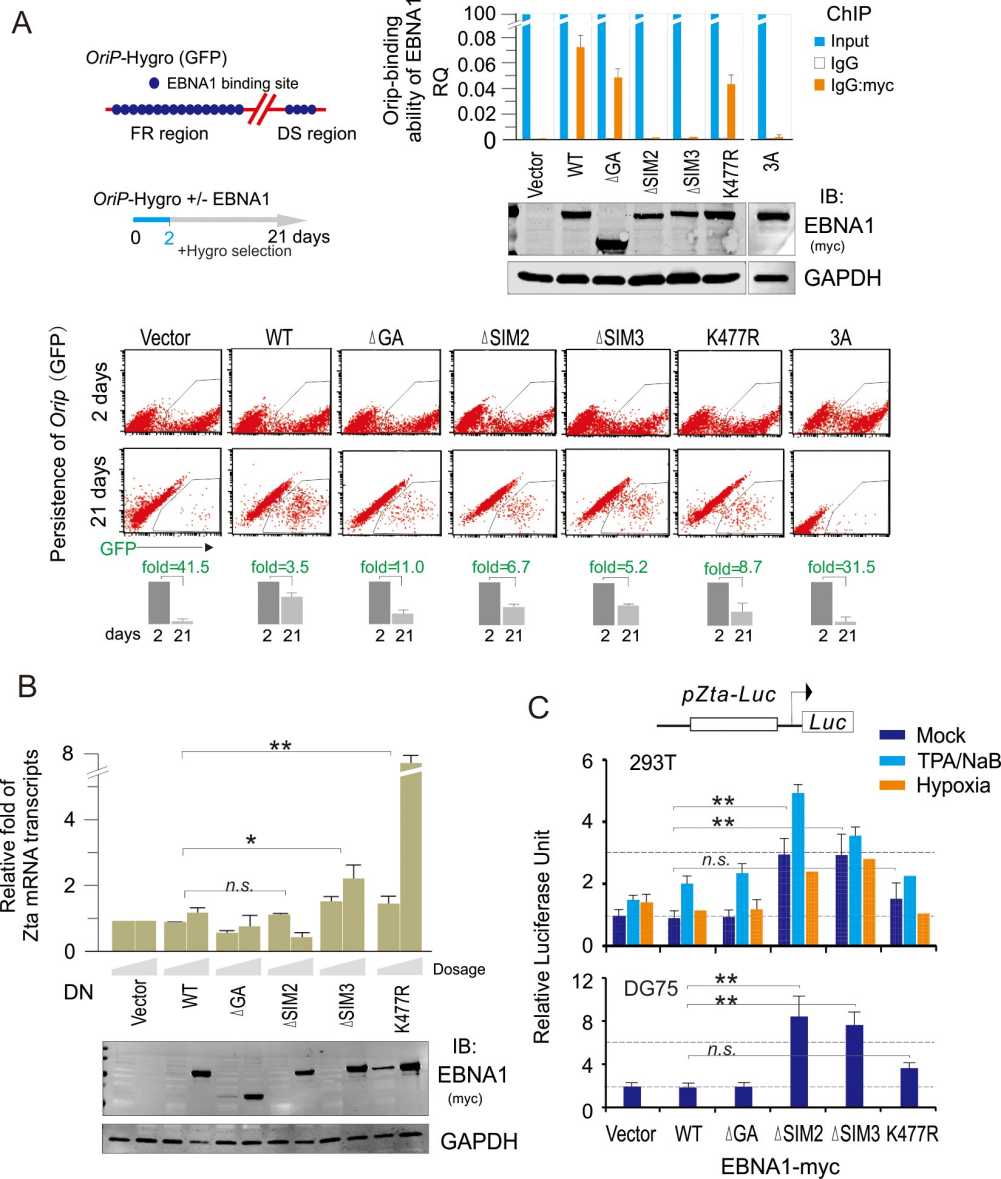
The EBNA1^SIM^ motif is important for DNA binding and maintenance of EBV *OriP* DNA. (**A**) Deletion of EBNA1^SIM^ reduces the DNA-binding ability of EBNA1 and the capacity of EBNA1-mediated *OriP* maintenance. HEK293 cells were transfected with *OriP*-GFP-Hygromycin plasmid together with vector encoding the WT or different mutants of myc-tagged EBNA1 or vector alone as indicated. The transfection efficiency was monitored by GFP expression. At 48 h post-transfection, cells were subjected to ChIP assays with normal mouse IgG or mouse anti-myc (9E10) antibodies followed by real-time quantitative PCR with *OriP* primers (*upper panel*). The level of *OriP* per transfection was compared to vector alone and shown as input. The results of *OriP*-binding ability of EBNA1 are presented after normalization with the protein levels of EBNA1. The expression of WT EBNA1 and its mutants were evaluated by IB assay and are shown in the figure (*right panels*). For flow-cytometry assays of EBNA1-mediated *OriP* maintenance, ten thousand of transfected cells were seeded at 2 days post-transfection and selected with 100 μg/ml Hygromycin for 21 days (*bottom panels*). The average of GFP-positive cells quantified from duplicate experiments is shown in the figure. (**B**) Dominant negative (DN) mutant of EBNA with SIM deletion reactivates EBV lytic replication. HEK293/Bac-EBV-GFP stable cells were transfected with different amounts of expression plasmids as indicated in the figure. At 48 h post-transfection, the relative level of Zta mRNA transcripts in transfected cells was detected by quantitative PCR. The expression levels of WT EBNA1 and its mutants were evaluated by IB assay and shown in the bottom panel. (**C**) Reporter assays of the Zta promoter in the presence of EBNA1 under different conditions. HEK293T or DG75 cells were individually co-transfected with a Zta promoter-driven Luciferase reporter (pZta-Luc) and vectors encoding the WT EBNA1-myc or its mutants (dGA, SIM2, SIM3, and K477R). At 24 h post-transfection, cells were exposed with 0.2% oxygen, TPA and sodium butyrate (NaB), or untreated as a control for overnight and then harvest for reporter assays. The results were presented by the RLU (relative luciferase unit) fold compared to pZta-Luc with vector alone. Data are presented as means±SD of three independent experiments. **p<0.01, *p<0.05; n.s. no significant.

Previous studies have shown that the DNA replication and segregation of EBV genome persistence during viral latency is dependent on the presence of the EBV *cis*-acting *OriP* and *trans*-acting viral protein EBNA1 [11,12,50]. To determine the role of EBNA1^SIM^ in EBNA1-mediated DNA replication and maintenance of the EBV episome DNA, we used a plasmid carrying the EBV *OriP* and a GFP reporter gene to mimic the EBV episome, and individually co-transfected with WT EBNA1 and its K477R, 3A or SIM-deleted mutants. The chromatin immunoprecipitation assay was carried out to assess the effect of K477R, 3A or SIM-deleted mutation on the ability of EBNA1 to bind to *OriP* DNA at 48 h post-transfection. The results showed that both 3A and SIM2 or SIM3 -deleted mutants dramatically blocked the ability of EBNA1 bound to *OriP* DNA, when compared with WT EBNA1 (Fig 4A, right panels). In contrast, both K477R or GA-deletion did not significantly impair the ability of EBNA1 bound to *OriP* DNA. This suggests that both intact SIM2 and SIM3 motifs of EBNA1 are required for the recognition and binding to *OriP*, albeit deletion of the SIM2 motif, but not SIM3, signficantly inhibits EBNA1 dimerization. To further investigate the effect of EBNA1 mutants on the stability of the *OriP* mini-genome during cell passage, equal amount of GFP-expressing HEK293 cells carrying *OriP* in the absence or presence of WT EBNA1 or its mutants were individually treated with hygromycin for three weeks and analyzed by flow cytometry. Consistently with previous findings, the results showed that the ratio of GFP-positive (*OriP* mini-genome) cells with 3A, SIM2 or SIM3-deleted EBNA1 were significantlly lower than that with co-expression of WT EBNA1 (Fig 4A, bottom panels). Unexpectedly, both K477R and the GA-deleted mutation also significantly reduced the maintenance of the *OriP* mini-genome, although they did not significantly impair the ability of EBNA1 bound to *OriP* DNA. These results indicate that the EBNA1^SIM^ motif is essential for the maintenance of the EBV episome.

Prvious studies demonstrated that EBNA1 depletion leads to an increase in lytic gene expression, suggesting that EBNA1 plays a role in transcriptional repression of lytic gene [2,51]. To determine whether the SIM motifs of EBNA1 are also involved in the inhibition of lytic replication, we transiently transfected different EBNA1^SIM^-related deficient mutants (as a dominat negative mutant) into HEK293 cells stably carrying the complete EBV genome with GFP label (viral genome), and then monitored the transcriptional level of Zta (the key activator of the EBV lytic life cycle) by quantitative PCR analysis. The results showed that only the SIM3-deletion or K477R mutation dramatically reactivated Zta transcription in a dose-dependent manner, when compared with WT EBNA1, ΔGA, or vector alone (Fig 4B). This strongly suggested that in addition to the maintenance of the viral episome, the EBNA1^SIM^ motif is also important for blocking lytic replication. To further determine whether the EBNA1^SIM^-dependent inhibition of lytic replication is due to directly silencing Zta expression, the Zta promoter-driven luciferase was used in a reporter assay in the 293T or DG75 cells with transiently presence of WT EBNA1, its SIM-deleted mutants or vector alone. The results showed that either SIM2 or SIM3 deletion enhanced the transcriptional activity of the Zta promoter when compared with that in the WT EBNA1 group (Fig 4C). Interestingly, under the stimulation with TPA and sodium butyrate or hypoxia, single deletion of SIM2 or SIM3 consistently induced higher transcriptional activities of the Zta promoter (Fig 4C), indicating that the EBNA1^SIM^ motif contributes to silencing Zta expression and blocking lytic replication.

### Deletion of EBNA1^SIM^ increases the SUMO1 to SUMO2 modification switch of STUB1 and its association with EBNA1

**Fig 5:**
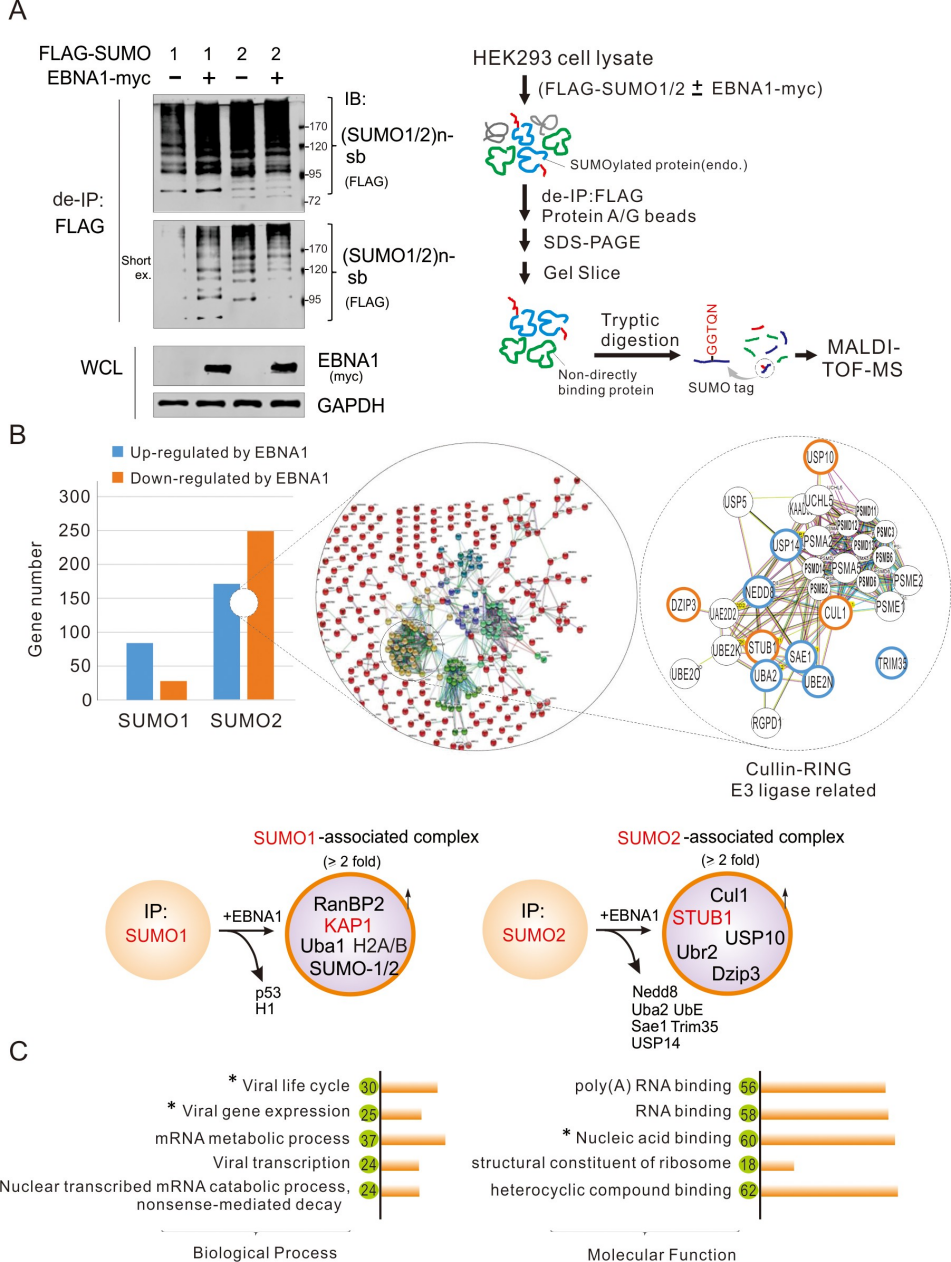
Ectopic expression of EBNA1 has global effects on cellular SUMO1 and SUMO2 modifications. (**A**) ENBA1 impairs SUMO1 and SUMO2-modified cellular proteins. Whole cell lysates from HEK293 cells co-transfected with expressing plasmids as indicated were analyzed by de-IP and IB with the indicated antibodies. The SUMO1/2-modified substrates [(SUMO1/2)n-sb] from immunoprecipitated complex are shown. The streamlined workflow for identification of profiles of SUMO1- or SUMO2-modified proteins is shown in the right panel. The heatmap and identified target proteins are shown in S3 Fig and S2 and S3 Tables. (**B**) Venn diagram of identified proteins with SUMO1/2 modification in the presence of EBNA1 by MALDI-TOF-MS. The number is obtained based on peptides of the proteins detected from mass spectrum (MS) with at least two-fold change from *panel A*. In the right panels, the hypothetical regulatory circuit of EBNA1-mediated SUMO2 modification of target substrates is shown, and the zoom-in of the core network with Cullin-RING E3 ligase-related molecules interconnected hubs is displayed. Blue nodes denote proteins significantly upregulated by EBNA1, and orange nodes are proteins significantly downregulated by EBNA1. The SUMO1- or SUMO2-target proteins by EBNA1 in the SUMO1/2-associated complex with more counts of peptide hits detected by MS are highlighted in the bottom panels. (**C**) The cellular pathways and functional clustering analysis of the identified proteins from SUMO2-associated complex mediated by EBNA1. The number of SUMO2-associated proteins is shown in the green circle. The related function of EBNA1 verified in this study is indicated using an asterisk.

To elucidate which proteins in the SUMO-modified complex are associated with the EBNA1^SIM^ motif, we first performed denatured IP assays to detect the cellular proteins with SUMO1/2 modification by transiently co-expressing exogenous SUMO1 or SUMO2 in the presence or absence of EBNA1 in HEK293 cells, followed by mass spectrum analysis (Fig 5A, right panel). The results of the immunoblotting analysis showed that the cellular proteins with SUMO1 modification were increased when EBNA1 was co-expressed, while less SUMO2-modified cellular proteins were observed when SUMO2 was co-expressed with EBNA1 (Fig 5A). Meanwhile, the results from mass spectrum analysis further revealed that the affinity of 84 out of 112 cellular proteins associated with SUMO1 was dramatically changed in the presence of EBNA1 (including RanBP2, KAP1, Uba1, H2A/B and SUMO1/2, which were up-regulated, and p53 and H1, which were down-regulated). In contrast, the affinity of 249 out of 424 cellular proteins associated with SUMO2 were impaired by EBNA1 (including Nedd8, Uba2, UbE, Sae1, Trim35, and USP14, which were down-regulated, and Cul1, STUB1, USP10, Ubr2, and Dzip3, which were up-regulated) (Fig 5B, left and bottom panels; S2 Table, S3 Table and S3 Fig). Intriguingly, the functional cluster analysis revealed that the SUMO2-associated protein complex targeted by EBNA1 is mainly related to the proteasome regulatory complex and ubiquitin-dependent Cullin-RING E3 ligase, indicating that EBNA1 may induce substrates for degradation via targeting their SUMO2-modified signaling. Further analysis of functional pathways showed that the SUMO2-modified proteins targeted by EBNA1 were mainly involved in regulating the viral life cycle, gene transcription, and expression, as well as mRNA metabolic processes, particularly those molecules for DNA and RNA binding (Fig 5C).

**Fig 6:**
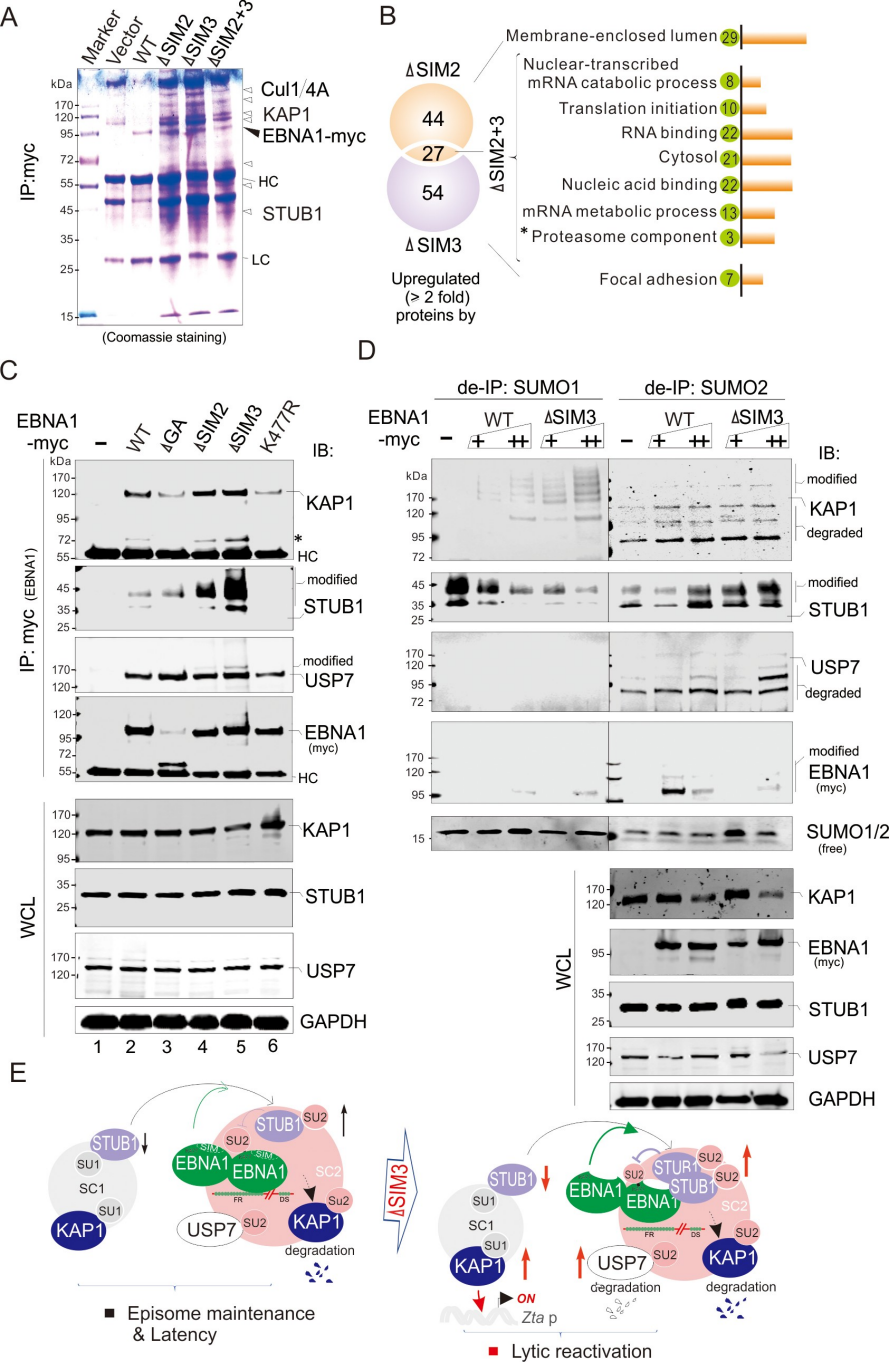
Deletion of EBNA1^SIM^ significantly increases SUMO2-modified STUB1 and its association with EBNA1. (**A**) Identification of cellular proteins that specifically associate with EBNA1^SIM^. Whole cell lysates from HEK293 cells individually transfected with vector alone or vectors encoding WT EBNA1 with myc tag, or its SIM-deleted mutants (ΔSIM2, ΔSIM3, and ΔSIM2+3), were subjected to IP with anti-myc antibodies. Equal amounts of EBNA1-precipitated proteins were subjected to SDS-PAGE separation followed by Coomassie staining or directly MALDI-TOF-MS analysis. The heatmap and identified target protein are shown in S4 Fig and S4 Table, respectively. HC, heavy chain; LC, light chain. The EBNA1 and its SIM-associated target proteins (STUB1, KAP1, Cul1, and Cul4A) with more counts of peptide hits detected by MS are highlighted in the *right panel*. (**B**) The functional cluster of EBNA1-associated molecules upon deletion of EBNA1^SIM^. The number of cellular proteins related to functional clusters of DNA/RNA binding, mRNA metabolism, transcription and translation process with significant change (≥ 2 fold) in the absence of EBNA1^SIM^ from *panel A* is shown in the green circles. The molecules related to the proteasome component are highlighted using asterisks. (**C**) Deletion of EBNA1^SIM^ significantly increases the association of STUB1 with EBNA1. HEK293 cells were transfected with plasmids expressing WT EBNA1 or its mutants (ΔSIM2, ΔSIM3, ΔSIM2+3, and K477R) with myc tag. At 48 h post-transfection, WCL were subjected to IB or IP with anti-myc antibody followed by immunoblotting as indicated. The asterisk denotes an uncharacterized protein band of KAP1. The modified protein bands of STUB1 and USP7 were highlighted. HC, heavy chain. (**D**) Deletion of EBNA1^SIM3^ enhances EBNA1-mediated STUB1 modification switch from SUMO1 to SUMO2, SUMO1-modified KAP1 and SUMO2-mediated degradation of USP7. HEK293 cells were individually transfected with different amounts of vectors expressing WT EBNA1, its ΔSIM3 mutant or vector alone. At 48 h post-transfection, WCL were subjected to directly immunoblotting or de-IP with antibodies specific against SUMO1 or SUMO2, followed by IB as the indicated antibodies. The degraded KAP1 or USP7 with SUMO2 modification is highlighted in the figure. (**E**) Schematic presents the role of EBNA^SIM^ in the regulation of SUMO1 and SUMO2 -associated complex. The SUMO1- and SUMO2-associated complex (SC1 and SC2) is shown in gray and pink, respectively. The effect of EBNA^SIM^ deletion is highlighted by red arrows in the right panels.

To further reveal which proteins are associated with the EBNA1^SIM^ motifs, we also individually performed co-IP assays by overexpressing WT EBNA1 and its SIM2 or SIM3 or both deleted mutants in HEK293 cells, followed by mass spectrum analysis (S4 Table, S4 Fig). Unexpectedly, the results from the gel with Coomassie staining clearly revealed a bunch of proteins significantly associated with EBNA1 upon the deletion of the EBNA1^SIM^ motif, which consistently appeared in the single or double deletion of SIM2 and SIM3 groups (Fig 6A). Among them, when compared with WT EBNA1, 44 proteins were exclusively related to SIM2 deletion, and 54 proteins only appeared in the SIM3 deletion group with a statistically significant 2-fold change, while 27 proteins were consistently detected in both single and double deletion of SIM2 and SIM3 groups (Fig 6B). Combined with our observation of a significant decrease of SUMO2-modified proteins upon EBNA1 co-expression (Fig 5A), these data indicates that the EBNA1^SIM^ motif is most likely involved in the regulation of SUMO2-mediated protein degradation. To further answer whether the EBNA1^SIM^-associated protein complex is indeed related to the SUMO2-modified proteins with EBNA1 expression, we also analyzed the biological function clusters of the identified proteins targeted by single or double deletion of SIM2 and SIM3 motifs. Similar to EBNA1-associated SUMO2-modified groups, the results showed that the EBNA1^SIM^-associated cellular proteins were also mainly related to DNA and RNA binding, gene transcription and expression (including KAP1), as well as proteasome degradation-related components (including STUB1, USP7, Cullin 1 and Cullin 4A) (Fig 6B). This further supports the notion that the EBNA1^SIM^ motif is essential for EBV episome maintenance and blocking the lytic gene expression.

To further confirm whether the deletion of EBNA1^SIM^ does impair the association of STUB1, KAP1, USP7 with EBNA1, we performed co-IP and immunoblotting assays with anti-myc antibodies in 293T cells ectopically expressing WT EBNA1 or its K477R or SIM-deleted mutant. The results showed that deletion of EBNA1^SIM^ did significantly enhance the association of STUB1 (both the ~35 kDa native and the ~45 kDa modified form) with EBNA1, while it slightly increased the association of KAP1 and its degraded form (~72 kDa), as well as a modified form (~170 kDa) of USP7 were observed, when compared with the WT group (Fig 6C, compared lanes 2 with 4 and 5). Interestingly, we also observed that K477R significantly abolished the association of EBNA1 with STUB1 and KAP1 but not USP7 (Fig 6C, compared lanes 2 with 6), which could be due to the exclusive localization of K477R mutant with chromatin DNA as a punctate dots, and indicating that SUMOylation of EBNA1 could be required for the interaction with STUB1 and KAP1.

To answer whether the EBNA1^SIM^-mediated modified forms of STUB1, KAP1 and USP7 are associated with the SUMO1 or SUMO2 signaling, we performed denature immunoprecipitation (de-IP) with endogenous SUMO1 or SUMO2 antibodies in HEK293T cells with different dosage expression of EBNA1 or its SIM3-deleted mutant. As shown in Fig 6D, deletion of the SIM3 motif resulted in an increase of SUMO1 modification of KAP1 and decrease of SUMO1 modification of STUB1 in the SUMO1-associated complex (SC1), and an increase of the SUMO2 modification of STUB1 and SUMO2-mediated degradation of USP7 in the SUMO2-associated complex (SC2). Consistent with the previous observation from immunofluorescent assays of the co-localization of EBNA1 with SUMO2 but not SUMO1, we also observed the appearance of SUMO2 instead of SUMO1 modified EBNA1 and a reduction in these forms upon the SIM3 deletion (Fig 6D). These results indicated that the EBNA1^SIM^ motif contributes to the inhibitory effects on the EBNA1-mediated STUB1 modification switch from SUMO1 to SUMO2, the SUMO1-modification of KAP1, and the SUMO2-mediated degradation of USP7 in the SC complex (Fig 6E).

### Hypoxia induces SUMO2-modified EBNA1 and the dissociation of EBNA1 with SUMO2-modified STUB1, KAP1, and USP7 for EBV lytic reactivation

**Fig 7:**
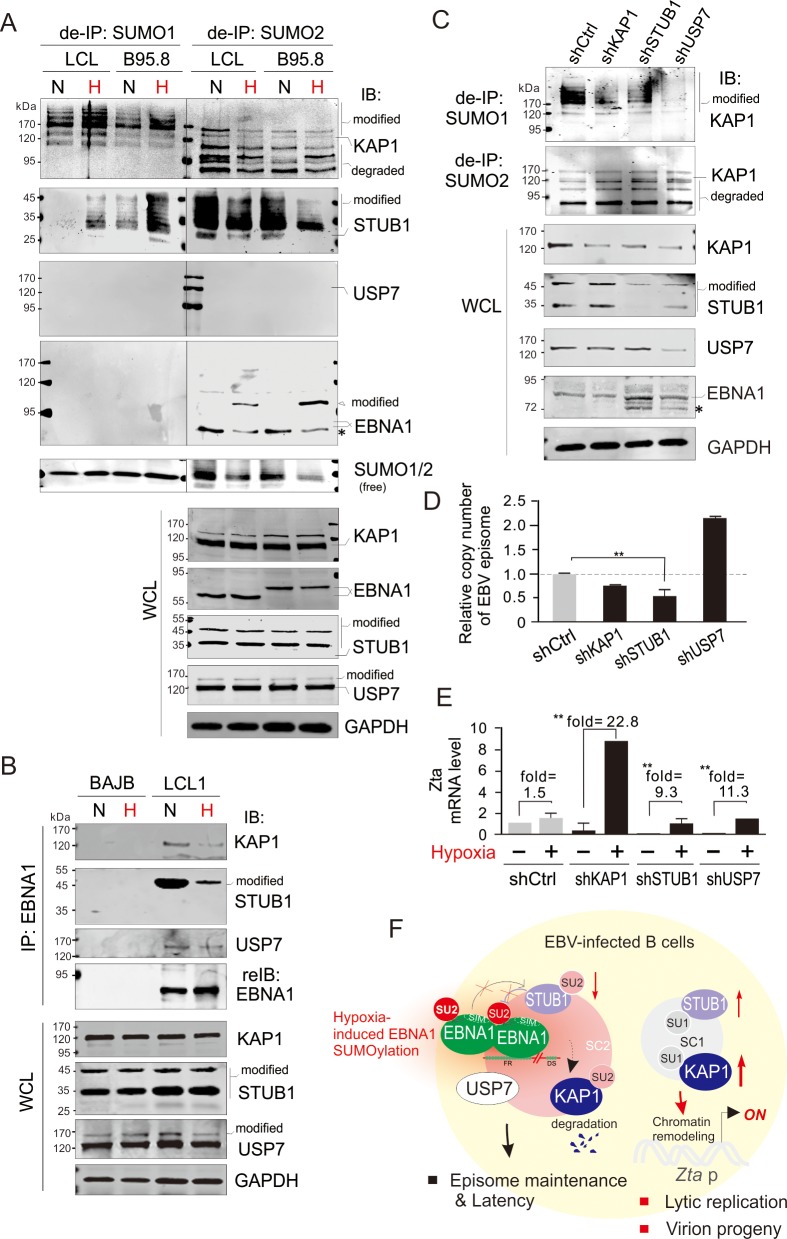
Hypoxia induces the dissociation of EBNA1 with STUB1, KAP1, and USP7 for reactivation of EBV lytic replication. (**A**) Hypoxia induces SUMO2-modification of EBNA1 and SUMO1-modification of both STUB1 and KAP1. Whole cell lysates (WCL) of the EBV-infected B lymphoma cells B95.8 and LCL1 treated with 21% (N) or 0.2% (H) oxygen for 48 h, were individually subjected to directly IB or de-IP with anti-SUMO1/2 antibodies followed by immunoblotting as indicated in the figure. The asterisk denotes an uncharacterized protein band of EBNA1. (**B**) Hypoxia reduces the association of EBNA1 with STUB1, KAP1, and USP7. The EBV-infected LCL1 and uninfected BJAB cells were treated with 21% (N) or 0.2% (H) oxygen for 48 h, respectively. WCL were individually subjected to IP with anti-EBNA1 antibody with the antibodies indicated in the figure. (**C**) STUB1 or USP7 knockdown reduces KAP1 expression and its SUMO1 modification. WCL of EBV-infected LCL1 cells with KAP1, STUB1 or USP7 knockdown were individually subjected to directly IB or de-IP with anti-SUMO1/2 antibodies followed by immunoblotting as indicated in the figure. The luciferase knockdown (shCtrl) was used as control. The asterisk denotes an uncharacterized protein band of EBNA1. (**D**) STUB1 knockdown significantly reduces the DNA copy number of EBV episome in latency. The episome DNA of EBV-infected LCL1 cells with STUB1, KAP1, USP7, or control knockdown from *panel C* were subjected to quantitative PCR analysis. The relative copy number of EBV episome is shown by comparison to the luciferase control knockdown. The asterisk indicates a significant difference after duplicate experiments. (**E**) STUB1 knockdown enhances the sensitivity of EBV lytic reactivation by hypoxia. The EBV-infected LCL1 cells with STUB1, KAP1, USP7 or luciferase control knockdown were individually subjected to treatments with 21% (N) or 0.2% (H) oxygen for 48 h. The relative levels of Zta mRNA transcripts in the cells with or without hypoxia treatment were quantified and are shown. (**F**) Schematic presents the role of EBNA1^SIM^-mediated SUMOylated proteins on the maintenance of EBV latency and lytic reactivation in response to hypoxic stress. The effect of SUMO2 modification of EBNA1 induced by hypoxia, and in turn dissociation of EBNA1 with STUB1, KAP1 and USP7, and increase of SUMO1 modification of both STUB1 and KAP1 for reactivation of EBV lytic replication is highlighted in red. The SUMO1- and SUMO2-modified protein complex (SC1 and SC2) is shown in gray and pink.

Previous studies showed that the SUMOylation of KAP1 is hypoxia-sensitive and hypoxia induces the dissociation of KAP1 from LANA for the KSHV latency to lytic replication switch [29], and hypoxia can induce EBV lytic replication [52]. We speculated that EBNA1-associated SUMOylation regulation of both STUB1 and KAP1 might also be occurring in response to hypoxic stress. To do this, a denatured IP with antibodies against the endogenous SUMO1 or SUMO2 was performed to verify the SUMOylation of KAP1 and STUB1 in the EBV-latently infected B cell lines B95.8 and LCL1. The results showed that the SUMO1-modified forms of both KAP1 and STUB1 were increased upon hypoxia treatment (Fig 7A). In contrast, SUMO2-modified forms of both KAP1 and STUB1 were consistently reduced by hypoxia to some extent, along with the increase of SUMO2-modified EBNA1 (Fig 7A), which was further confirmed by the observation that there was an increase in the co-localization of endogenous EBNA1 with SUMO2 instead of SUMO1 under hypoxic stress (Supplementary S5 Fig). Unexpectedly, distinct from the ectopic expression of EBNA1 in HEK293 cells, we did not observe SUMO1/2 modification of USP7 in any of the EBV-infected B cells (Fig 7A). Consistently, the interaction of EBNA1 with the SUMO2-modified STUB1 and KAP1, but not USP7, was dramatically reduced in hypoxia, when compared with the interaction in normoxia (Fig 7B). Given the fact that deletion of SIM3 motif enhances the interaction of EBNA1 with STUB1, while it is significantly blocked by mutation of Lysine 477 (K477R) (Fig 6C), indicating that the SUMO2 modification of endogenous EBNA1 may physiologically compete with alternative modification at K477 to prevent interaction of EBNA1 with STUB1 under hypoxic condition.

To further answer which protein in the EBNA1 complex is important for the association with EBNA1, we attempted to specifically knocked down the expression of STUB1, KAP1 or USP7 in LCL cells by using different small hairpin RNA target sequences (S6 Fig), and carried out denature immunoprecipitation and immunoblotting assays. In addition to a lower level of STUB1 expression was appeared upon USP7 knockdown (which suggests that USP7 stabilizes STUB1), the results consistently showed that inhibition of STUB1 or USP7 led to decreased expression of KAP1 and its SUMO1 (not SUMO2) modification, along with slightly increased protein level of EBNA1, albeit the effect of STUB1 knockdown on EBNA1 is much higher than that of USP7 knockdown (Fig 7C). This indicates that the association of EBNA1 with STUB1 is critical for EBNA1-mediated latency and in turn the silencing of lytic replication, and the chromatin remodeler KAP1 is one of STUB1 downstream targets. To this end, the relative copy number of the EBV episome in LCL cells with STUB1, KAP1, USP7, or control knockdown was analyzed. The results showed that knockdown of STUB1 instead of KAP1 dramatically reduced the DNA copy number of the EBV episome, while the USP7 knockdown increased it (Fig 7D). In agreement with this speculation, upon the stimulation of hypoxia for EBV lytic reactivation, we consistently detected higher levels of Zta transcription in the STUB1 or KAP1 knockdown cells than that in the luciferase control knockdown cells, although a much higher response to hypoxia was observed in the cells with KAP1 knockdown compared with the STUB1 knockdown (Fig 7E). However, a higher response to hypoxia was also unexpectedly observed in the USP7-knockdown cells than in the luciferase control knockdown cells (Fig 7E). This further confirms that the EBNA1^SIM^-mediated SUMOylated proteins including STUB1, KAP1 and USP7 are essential for the maintenance of viral latency to block lytic replication.

## Discussion

Since SUMOylation has been discovered to be involved in the regulation of many cellular biological processes, it is not surprising that viruses have evolved different strategies to manipulate the host SUMO signaling to their own advantage. Our previous studies showed that the LANA protein encoded by KSHV contains a SUMO2-interacting motif, which is essential for the latent viral infection and is also involved in the dissociation from SUMO2-modified KAP1 in response to hypoxic stress [29]. The EBNA1 encoded by EBV, as a homolog of LANA, was also shown to play an important role in maintaining the stability of EBV episome and regulating the host and viral gene transcriptions [2,51]. In this study, we found that EBNA1 contains a SIM motif (EBNA1^SIM^) that is required for the EBNA1-mediated inhibitory effects on SUMO1-modified KAP1, SUMO2-modified STUB1, and SUMO2-mediated degradation of USP7. Deletion of the EBNA1^SIM^ motif leads to the functional loss of both EBNA1-mediated viral episome maintenance and lytic gene silencing (Fig 6E). Importantly, the hypoxic stress induces SUMO2-modification of EBNA1 in EBV-infected B cells, and results in the dissociation of EBNA1 with STUB1, KAP1 and USP7 to increase SUMO1-modification of both STUB1 and KAP1 for reactivation of lytic replication (Fig 7F).

KAP1, also named as Trim28, is well characterized as a universal corepressor of the KRAB-domain containing zinc finger proteins in the human genome. KAP1 itself cannot bind DNA directly and recruits or coordinates the assembly of several chromatin-remodeling proteins including NCoR1 and HP1. Emerging evidence suggests that KAP1 is involved in the regulation of herpesvirus latency and lytic reactivation. In addition to the phosphorylation of KAP1 at S824 that occurs in the lytic replication of both KSHV and EBV [53,54], we observed that SUMO2 modification of KAP1 is also shut off for the lytic reactivation of EBV from latency in response to hypoxic stress, which is similar to what we previously observed during KSHV reactivation [29]. Interestingly, our finding provides the evidence for the first time that the switch of KAP1 from SUMO2 to SUMO1 modification facilitates the reactivation of EBV lytic replication. Strikingly, a switch of STUB1 from SUMO2 to SUMO1 modification is also observed in the reactivation of EBV lytic replication. In the view of the fact that the STUB1 knockdown results in the inhibition of KAP1 expression, this indicates that STUB1 could be one of the upstream regulators of KAP1, and the KAP1 modification switches from SUMO2 to SUMO1 could be due to the STUB1 modification switch, which in turn subtly regulates the switch of EBV from the latent to the lytic life cycle.

STUB1 (also named as CHIP, for C-terminus of HSP70 interacting protein) is an E3 ubiquitin ligase widely expressed in mammalian cells and tissues. Under stress conditions, STUB1 has been shown to act as a chaperone HSP90/70-assisted E3 ligase primarily involved in ubiquitylation and degradation of unfolded substrates for protein quality control [55,56]. STUB1 is not only mechanistically linked to clearance of unfolded modified proteins in different types of cancer [57], but also controls the stability of protein kinases involved in different aspects of cell physiology [58,59], suggesting that STUB1 has a dual function as a regulator of protein homeostasis and an ON/OFF switch for cell signaling. In this study, we further reveal for the first time that STUB1 undergoes SUMO1 and SUMO2 modification switch and is targeted by the EBNA1^SIM^ motif to be involved in the regulation of EBV latency maintenance. In addition, STUB1 not only acts as a key component of the EBNA1^SIM^-associated complex for viral latency, as the knockdown of STUB1 also leads to the down-regulation of KAP1 expression and in turn increases the sensitivity of LCL cells to respond to hypoxic stress, and as a consequence go through the viral lytic replication. Previous studies showed that stress signals elicited by proinflammatory cytokines such as IL-6 and also by LPS could induce the degradation of Foxp3 (the key Treg cell transcription factor) through the E3 ubiquitin ligase of STUB1 [60]. In this regard, our data is also interesting for showing that the SUMO-modification of STUB1 is involved in the response to hypoxic stress.

Although it has been previously demonstrated that EBNA1 is important for the replication and mitotic segregation of the EBV episomes through direct interaction with the *OriP* sequence [61,62], the posttranslational regulation utilized by EBNA1 to maintain EBV latent infection remains largely unclear. Our studies reveal that EBNA1 contains two SIM motifs, SIM2 and SIM3, which are located within the DNA-binding and dimerization domain of EBNA1 that is a functional structure for binding to the viral genome *OriP*. Although the 3D structure analysis reveals that both SIM2 and SIM3 are located close to the interacting edge of two EBNA1 monomers, our findings show that only the deletion of SIM2 strikingly interrupts the dimerization of EBNA1, indicating that these two SIM motifs engage with different partners in regulating EBNA1 functions. Their simultaneous deletion leads to loss of EBNA1-mediated DNA-binding ability, *OriP*-mimic episome stability and inhibition of the Zta promoter. This suggests that the SUMOylation mediated by EBNA1^SIM^ is essential to maintain the stability of the episomal DNA during EBV latency.

To reveal the profiles of host cellular proteins interacting with EBNA1^SIM^, we have performed tandem affinity purification-tagging approaches under a native or denatured condition. Unexpectedly, although USP7 was previously identified as a specifically interacting protein of EBNA1 was previously identified [25], and the assembly of EBNA1 on *OriP* elements is decreased by USP7 silencing [63], the affinity of UPS7 to EBNA1 does is not significantly different once the EBNA1^SIM^ motif is deleted. In contrast, we observed that EBNA1 induces the SUMO2 modification of USP10 instead of USP7, albeit the efficiency with which USP10 cleaves linear ubiquitin is much lower than that of USP7 according to previous reports [64,65]. However, the related mechanisms of EBNA1-mediated SUMO2 regulation of USP10 need further investigation. In addition, USP7 is known as an H2B deubiquitinase and is often recruited by EPOP to chromatin for activating gene transcription [66]. In the view of the facts that EBNA1 is able to recruit USP7 to the EBV latent origin for DNA replication [63], and STUB1 antagonizes USP7 to ubiquitinate Foxp3 for inflammatory response [67], it is not surprising to observe that the SUMO2-mediated degradation of USP7 is induced by the deletion of the EBNA1^SIM^ motif, along with the increase of SUMO2-modified STUB1. This could partly explain why the deletion of EBNA1^SIM^ motif leads to the activation of viral lytic genes, as observed in the Zta promoter-driven luciferase reporter assays.

Consistent with previous reports [68], our study also reveals that absence of the GA-repeat region of EBNA1 will result in loss of EBNA1-mediated *OriP*-based plasmid maintenance in long-term passages, albeit its dimerization and DNA-binding ability are not significantly changed. Here, we highlight the fact that both the K477 SUMOylated residue and the SIM motif of EBNA1 encoded by the different EBV strains are highly conserved [69], which indicates that EBNA1-mediated SUMOylation regulation is critical. The distribution of both SIM2 and SIM3 at the surface of EBNA1 and in the dimerization region further supports our speculation. In addition, when the K477 residue was mutated, the association of EBNA1 with STUB1 was dramatically disrupted, and resulted in an exclusively co-localization of EBNA1 with chromatin DNA as punctate dots, indicating that SUMO modification on K477 plays a critical on the function of EBNA1. This is further supported by the lower persistence of the *OriP*-mimic episome when K477R mutated and the hypoxia-induced SUMO2-modification of EBNA1 for lytic reactivation. It is also worth mentioning that only EBNA1 co-localization with SUMO2 instead of SUMO1 as punctate dots is observed in the subcellular compartment, and deletion of SIM3 motif leads to reduction of the EBNA1 co-localization with SUMO2 within nuclear compartment and the poly-SUMO2 modification of EBNA1 *in vitro*. This indicates that the SIM3 motif contributes to SUMO2 modification of EBNA1 *in vitro* and *in vivo*.

Although EBNA1 was found to induce the degradation of promyelocytic leukemia (PML) nuclear bodies (also called ND10) in both NPC and gastric carcinoma cells [70,71], its effect remains unclear in B-lymphoma cells. Consistently, we did also observe that the punctate dots of SUMO1 or SUMO2 were greatly reduced by EBNA1 co-expression. In addition, why the interaction of EBNA1 and USP7 is required for the EBNA1-mediated degradation of PML was also unclear. Our findings that the ubiquitin E3 ligase STUB1 is recruited by the EBNA1^SIM^ for inhibition of the SUMO1-modified KAP1 (a SUMO E3 ligase) provides a potential explanation. The SUMO2-mediated degradation of USP7 observed under the ectopic expression of EBNA1 in HEK293 cells, but not when LCL cells express endogenous levels of EBNA1, suggesting that USP7 could be a key balance molecule to establish latency from primary infection.

Previous studies have shown that the DNA binding and dimerization domains of EBNA1 are mapped between residues 459 and 607 at the carboxyl terminus, and the crystal structure of EBNA1 reveals a glycine-rich region that is responsible for both direct DNA recognition and RNA binding [72]. EBNA1 can act as a strong RNA binding protein, interacting with diverse substrates *in vitro*, including the EBV-encoded RNA polymerase III transcript EBER1 and the HIV-encoded transactivation response (TAR) element. Consistently with a previous report [72], from the results of proteomic analysis of EBNA1 co-expression with SUMO1/2 or deletion of EBNA1^SIM^, we also observed that many DNA and RNA binding proteins are co-purified with EBNA1 through its SUMO-signaling or SIM motifs. In particular, a subset of heterogeneous ribonucleoproteins (hnRNPs) was isolated with EBNA1 but not with the negative control. Therefore, the RNA-mediated association of EBNA1 with specific hnRNPs may also reflect a tendency of EBNA1 to interact with the same RNA molecules or sequences as these hnRNPs [10].

Taken together, our findings identify STUB1 as an important signaling node that dynamically links KAP1 to USP7, is targeted by EBNA1 through its SIM motif, and thereby controls the EBV latency and lytic replication in response to hypoxic stress. The discovery of the intersection between SUMO1 and SUMO2 modification switch of both STUB1 and KAP1 sheds new light on the regulation of EBV latency and lytic replication, which provides a potential therapeutic target against EBV-associated cancers.

## Materials and methods

### DNA constructions

The plasmids pA3M-EBNA1 (pA3F-EBNA1) expressing full length EBNA1with myc tag was individually constructed by ligation EBNA1 fragment from pBS-EBNA1 (AJ507799 EBNA1 sequence, direct-site mutation with ATG-*Bam*HI and TGA-*Eco*RI) into pA3M (or pA3F) vector digestion with *Bam*HI and *Eco*RI. The SIM-deleted (ΔSIM1, ΔSIM2, ΔSIM3, and ΔSIM2+3), SIM2-M1, SIM2-M2, SIM2-4A SIM3-3A and K477R mutants of EBNA1 were constructed by PCR-directed site mutation based on pA3M-EBNA1-ΔGA (kindly provided from Erle Robertson at University of Pennsylvania) as template, respectively. The SIM-deleted and K477R mutants of full length EBNA1 were individually generated by the corresponding mutant fragment from pA3M-EBNA1-ΔGA with *Apa* I or *Basu*36I and *Sac*II digestion and replaced the EBNA1 C-terminal region of pA3M-EBNA1. The EBNA1 DBD (441–619 aa) truncated mutant was inserted into pGEX-2TK vector by PCR amplicon with *Bam*HI and *Eco*RI digestion. The *OriP*-GFP plasmid with hygromycin was generated from *OriP*-GFP-EBNA1 plasmid (a kindly gift from Paul M. Lieberman at Wistar Institute) by *Sac*I digestion to remove EBNA1 DNA fragment and re-ligation. The plasmids pGL3-Zta (pZta-luc) was generated by PCR amplicon of Zta promoter region (-1000 to 1 bp) from B95.8 EBV genome DNA as template with *Kpn*I and *Bgl*II digestion and ligation into pGL3 vector. Cherry-SUMO1 was generated by the SUMO1 fragment from FLAG-SUMO1 plasmid inserted into pmCherry C1 vector with *Kpn*I and *Bam*HI digestion. Cherry-SUMO2 was generated by the SUMO2 fragment from FLAG-SUMO2 plasmid inserted into pmCherry C1 vector with *EcoR*I and *Bam*HI digestion. The plasmids HA-Ubc9, FLAG-SUMO1, and FLAG-SUMO2 were stored in the lab[29].

### Reagents and antibodies

Antibodies to EBNA1 (sc-57719, Santa cruz), FLAG (M2, Sigma), SUMO1 (Y299, Abcam), SUMO2/3 (for IB/IF, EPR4602, Abcam; for IP, fsc-393144, Santa cruz), STUB1 (EPR4447, Abcam), KAP1 (20C1, Abcam), GST (12G8, M20007, Abmart) and GAPDH (G8140-01, US Biological) were used according to the manufacturers specifications. The monoclonal antibody anti-myc (9E10) and HA (12CA5) were prepared from hybridoma cultures stored in the laboratory. Tetradecanoyl Phorbol Acetate (TPA) was purchased from Sigma and sodium butyrate from J&K Corporation. Proteasome inhibitors PMSF, Leupeptin, Aprotinin, Pepstatin A and Puromycin were purchased from Amresco.

### Cell culture and transfection

EBV positive cell line B95.8 was purchased from ATCC and EBV-transformed primary B cell line LCL1 was kindly provided by Xiaozhen Liang from Shanghai Pasteur Institute of CAS. All the EBV positive and negative (DG75, stored in the lab) B-lymphoma cells were maintained in RPIM1640 (Hyclone) medium with 10% fetal bovine serum (FBS) and 1% penicillin and streptomycin (Gibco-BRL). 293T cells were maintained in DMEM medium supplemented with 10% FBS) and 1% penicillin and streptomycin (Gibco-BRL). All cell lines were incubated at 37°C in a humidified environmental incubator with 5% CO2. For transfection, 293T cells were cultured for 24 h before transfection with cell confluence reaching 60–70%, and then transfected with plasmids DNA and polyethyleneimine (PEI) mixture at a ratio of 1μg DNA/3μl PEI (1mg/ml). DG75 cells transfection was performed with Lonza-4D nucleofector system in an optimized program, CA137.

### Protein expression, purification, and in vitro pull-down assays

Overnight starter cultures (50 ml) of BL21 (DE3) transformed with plasmid expressing GST or GST-fused EBNA1, or His-fused SUMO1 or SUMO2 protein were individually inoculated 500 ml of Luria Broth (LB) culture medium with specific antibiotic and grown at 30°C to a density of appropriately 0.6 optical density at 600nm. After 1 mM isopropylthiogalactopyranoside (IPTG) induction at 30°C for 4 h, the bacteria were collected and sonicated in lysis buffer (20 mM Tris-HCl pH 8.0, 100 mM NaCl, 0.5% NP40, 1 mM EDTA, 1 M DTT, 5% Sarkosyl and the protease inhibitor cocktail). Recombinant proteins GST, GST-EBNA1-DBD (WT, ΔSIM2, SIM2-4A, ΔSIM3, SIM3-3A, or K477R) was purified by Glutathione Sepharose chromatography according to manufacturer’s instruction (Amersham Biosciences), and His-SUMO1 and His-SUMO2 proteins were purified by Ni^2+^-NTA agrose chromatography (Qiagen). For pull-down assay, cell extracts were individually incubated with His-fusion proteins loaded on beads for 3 h at 4°C in NETN binding buffer (50 mM Tris-HCl pH 7.5, 100 mM NaCl, 10 μm ZnCl_2_, 10% glycerol, freshly supplemented with 0.1 mM Dithiothreitol and protease inhibitors). After washing, bound proteins were eluted with SDS sample buffer and analyzed by gel electrophoresis followed by Coomassie staining or immunoblotting with specific antibodies as described previously [29].

### Circular dichroism (CD) spectroscopy

The CD spectroscopy was conducted as described previously [73]. Briefly, the CD spectra were carried out in a Jasco spectropolari-meter (model J-815; Jasco, Inc., Easton, MD, USA) with sample holder in a 0.1 cm path length cell and a protein concentration range of 15–20μM. Spectra were collected using a 5-nm bandwidth with a 5-nm step resolution from 195 to 350 nm at room temperature. The spectra were corrected by subtraction of 0.1M Tris pH8.0. The melting curve was smoothed.

### Immunoprecipitation and immunoblotting

Immunoprecipitation (IP), denatured IP and immuno-blotting assays (IB) were performed as described previously [29,74]. The interesting proteins in the membrane were scanned and analyzed by the Odyssey Infrared scanner and its software (Li-Cor Biosciences).

### In vitro SUMOylation assay

All purified recombinant proteins SUMO-1/2, Aos1/Uba2 and Ubc9 proteins were purchased, and in vitro SUMOylation assay was performed according to manufacturer’s instruction (Shanghai Chairmade Inc., China), GST-EBNA1 fusion protein was purified from *Escherichia coli* BL21 (DE3) as described previously. Briefly, the reaction system of In vitro SUMOylation assay was incubated for 3 h at 37°C in a 100 μl volume containing 100 nM Aos1/Uba2, 1.5 μM Ubc9, 10 μM mature SUMO-1/2, 50 mM Tris, 5mM MgCl2, 2mM ATP, pH 7.5, and 0.5 μM of purified GST-EBNA1. Reaction products were directly quenched with SDS loading buffer, and subsequently analyzed by SDS-PAGE and immunoblotting with GST antibody.

### Immunofluorescence assays

Suspension cells (B95.8 and LCL1) were harvested and washed with ice-cold PBS twice by centrifuge at 300g for 5 minutes, then fixed in 4% paraformaldehyde. After fixation, cells were washed three times in PBS. The cells were resuspended in PBS containing 0.2% fish skin gelatin (G-7765; Sigma) and smeared on the coverslips. The followed the primary and secondary antibodies staining and DNA counterstained with DAPI were performed as the described previously [75].

### Dual-luciferase reporter assay

Luciferase reporter plasmid pGL3-ZTAp-luc was used to detect the effect of SIMs of EBNA1 on viral gene expression. Renilla luciferase was used as a control to normalize the transfection efficiency. Relative luciferase activity [RLU] was expressed as fold changes relative to the reporter construct alone. Assays were performed in triplicate.

### Chromatin immunoprecipitation

Approximate thirty million of 293T cells individually transfected with wild type EBNA1 and its mutants in the presence of *OriP*-GFP-Hygromycin plasmid at 48 h post-transfection, were cross-linked with 1.42% (v/v) formaldehyde. The following protocols were performed as described previously [75]. The antibodies against EBNA1 (sc-57719, Santa cruz) was used for chromatin immunoprecipitation. Purified DNA was amplified by quantitative PCR with the specific *Orip* primers as shown in S1 Table.

### Protein identification of mass spectrometry and data analysis

Twenty micrograms of total bound proteins were separated on a 4−15% precast gel (BioRad). The gel was Coomassie stained. Protein standard bands served as a guide for the excision of gel slices of various molecular weight size ranges. The excised gel pieces were subjected to washing with 100 μl of 50 mM ammonium bicarbonate in 50% acetonitrile, and dehydrated in acetonitrile followed by solvent removal using a vacuum centrifuge. Samples were then swollen in 100 μl of a digestion buffer containing 50 mM ammonium bicarbonate, 5 mM calcium chloride (50 μl), and 12.5 ng/ml of trypsin for overnight digestion. Peptides were extracted into 20 mM ammonium bicarbonate (100 μl) followed by two separate extractions into 100 μl of water/acetonitrite/formic acid (10:10:1, v/v/v). Extracted peptides were resuspended in 10 μl of 5% acetonitrile and 0.1% trifluoroacetic acid, run on a matrix-assisted laser desorption/ionization-TOF (MALDI-TOF) mass spectroscopy (Applied Biosystems).

Peptide matches were identified by MALDI-TOF mass spectroscopy for molecular weight determination and MALDI-TOF/TOF for sequence information, and nanoLC/Qstar-XL (Applied Biosystems) analysis. The data were analyzed with GPS explorer/Analyst QS software and were searched with Mascot software (Matrix Science Ltd.) against the National Center for Biotechnology Information database (NCBI). The proteins with MudPIT score with a cut-off above 40 were selected for analysis. Then enrichment analysis was fulfilled between the core and extended network and all the current available pathway and function categories. The most significant pathways and function categories were picked out on the basis of the enrichment P-value. The enrichment P-value is calculated on the basis of the hypergeometric distribution in this study.

### Quantitative PCR

Total RNA was extracted by using TRIzol (Shanghai Yeasen Biotech Co., Ltd, 10606ES60) and reverse transcribed into cDNA with Goldenstar RT6 cDNA synthesis (Beijing Tsingke Biotech Co., Ltd). The cDNA was amplified in a 20 μl total volume containing 10 μl SYBR green, 0.5 μl each primer (10 μM), 8 μl H_2_O, and 1 μl cDNA. A melting-curve analysis was performed to verify the specificities of the amplified products. The values for the relative levels of change were calculated by the threshold cycle (ΔΔCT) method, and samples were tested in triplicates. The primers used for real-time PCR were shown as in S1 Table.

### RNA interference

The oligo sequences against STUB1 (target sequence 1: 5’-GUGAGAGGGAGCUGGAAGA-3’; target sequence 2:5’-AGGCCAAGCACGACAAGUA-3’)[76], USP7 (Target sequence 1: 5’-CCCAAAUUAUUCCGCGGCAAA-3’; target sequence 2:)[63], KAP1 (5‘-GCAUGAACCCCUUGUGCUG-3’) and non-specific control sequence (5’-UGCGUUGCUAGUACCAAC-3’) were used as previous reports[29]. The DNA oligo of short hairpin RNA sequences (shRNA) targeted to STUB1, KAP1 and USP7 were inserted into the pGIPz vector followed the Clonetech manufacturer’s instructions. The pGIPz vector carrying different shRNAs were co-transfected with lentivirus packaging plasmids into HEK293T cells for 48 h to generate lentiviruses, respectively. The shRNA-packaged lentiviruses were individually traduced into 293T cells and LCL1 cells, followed by treatment with 2 μg/ml of puromycin. The immunoblotting analysis with STUB1, KAP1 and USP7 antibodies was individually used to verify the efficiency of RNA interference.

### Statistical analysis

The statistical analysis was performed by using SPSS software. The experimental and control groups were assessed by student’s *t*-test for single comparisons. *P* values less than 0.05 were considered to indicate statistically significant differences.

## Supporting information

S1 Table List of DNA oligonucleotides used in this study. (DOCX)

S2 Table List of SUMO1-associated proteins in the presence of EBNA1 identified by Mass Spectrum analysis with significantly difference (≥ 2 fold). (DOCX)

S3 Table List of SUMO2-associated proteins in the presence of EBNA1 identified by Mass Spectrum analysis with significantly difference (≥ 2 fold). (DOCX)

S4 Table List of EBNA1^SIM^-associated proteins identified by Mass Spectrum analysis with significantly difference (≥ 2 fold). (DOCX)

S1 Fig EBNA1 interacts with SUMO1 and SUMO2. HEK293 cells were co-transfected with expression plasmids as indicated in the figure. At 48 post-transfection, whole cell lysates were subjected to immunoprecipitated (IP) and immunoblotting (IB) as indicated. The position (>170 kDa) of EBNA1-interacting SUMO1 or SUMO2 modified substrates [(SUMO1/2)n-sb] is highlighted.
(TIF)

S2 Fig The intact SIM2 or SIM3 motif of EBNA1 enhances the affinity of EBNA1 with SUMOylated Ubc9. HEK293T cells were co-transfected with expression plasmids as indicated. Whole cell lysates (WCL) were harvested at 48 h post-transfection, and subjected to co-IP and IB as indicated. The relative density (RD) of EBNA1-binding Ubc9 and Ubc9-SUMO is quantified and shown on the right panel.
(TIF)

S3 Fig Heatmap of the SUMO1/2-associated cellular proteins in the presence and absence of EBNA1 identified by MALDI-TOF-MS analysis. Related to Fig 4A.
(TIF)

S4 Fig Heatmap of the cellular proteins associated with full length EBNA1 and its mutants identified by MALDI-TOF-MS analysis. Related to Fig 6A.
(TIF)

S5 Fig Hypoxia increases the co-localization of EBNA1 with SUMO2. LCL1 cells were subjected to hypoxia (0.2% oxygen) treatment for overnight. Endogenous EBNA1, SUMO1, and SUMO2 were individually stained by EBNA1 (green) and SUMO1/2 (red) antibodies. The profile of EBNA1 and SUMO1/2 immunofluorescence were quantified and shown on the right panels. SUMO2 co-localization with EBNA1 was highlighted by the arrows and enlarged at the bottom panels.
(TIF)

S6 Fig Whole cell lysate of 293T (**A**) or LCL (**B**) cells with KAP1, STUB1 or USP7 knockdown were individually subjected to immunoblotting (IB) with the indicated antibodies. The luciferase knockdown (shCtrl) was used as control.
(TIF)
