# Supplementary material for: STUB1 is targeted by the SUMO-interacting motif of EBNA1 to maintain Epstein-Barr Virus latency
Source: PLoS Pathog. 2020 Mar 16;16(3):e1008447. doi: 10.1371/journal.ppat.1008447 (PMC7105294; doi:10.1371/journal.ppat.1008447)
Supplement: S1 Table — (DOCX) [file ppat.1008447.s001.docx]

**Table S1**. List of DNA oligos used in this study.

| Primers | Oligo sequence | Targets |
| --- | --- | --- |
| EBNA1-F1 | 5'-CCGACTCGGATCCGCCATGTCTGACGAGGGG-3' | EBNA1 full length PCR with *Bam*HI and *Eco*R I |
| EBNA1 R1 | 5’-TGTG GAATTC TGCAGATA-3’ |  |
| EBNA1 1254F | 5’-CCAAGAAGGTGGCCCAGATGGTGAG-3' | For wild type EBNA1-myc and its mutant construction with *Apa* I and *Xba* I |
| EBNA1 myc-R | 5’-CCTCTAGACTAGCTAGTTAACCGGT-3’ |  |
| EBNA1 Trunc-S | 5’-TCGGGATCC-GACCCAGGAGAAGGCCCAA-3 | For EBNA1 truncation construction with *Bam*HI and *Eco*RI |
| EBNA1 Trunc-AS | 5’-GGAATTC-CGCGGCAGCCCCTTCCACCATA-3’ |  |
| dSIM1-S | 5'- GGAGAAAAGAGGCCCAGGCCACCGCGCAGGCCCCCT -3' | For SIM1 deletion of EBNA1 (AJ507799) |
| dSIM1-AS | 5'-AGGGGGCCTGCGCGGTGGCCTGGGCCTCTTTTCTCC -3' |  |
| dSIM2-S | 5'-AAGGAACTTGGGTCGCCGGTTCCCTTTACAACC-3 | For SIM2 deletion of EBNA1 (AJ507799) |
| dSIM2-AS | 5'-GGTTGTAAAGGGAACCGGCGACCCAAGTTCCTT-3' |  |
| SIM2-M1 S | 5'-GGAACTTGGGTCGCCGGTGCGGCCGCATATGGAGGTAGTAAGACC-3' | For SIM2-M1 site mutation |
| SIM2-M1 AS | 5'-GGTCTTACTACCTCCATATGCGGCCGCACCGGCGACCCAAGTTCC-3' |  |
| SIM2-M2 S | 5'- CGTATATGGAGGTGCTGCAGCGTCCCTTTACAACC -3' | For SIM2-M2 site mutation |
| SIM2-M2 S | 5'-GGTTGTAAAGGGACGCTGCAGCACCTCCATATACG-3' |  |
| dSIM3-S | 5'-AGCCCGCTCCTACCTGCAATTGCAGCTTTGAC-3 | For SIM3 deletion of EBNA1 (AJ507799) |
| dSIM3-AS | 5'-GTCAAAGCTGCAATTGCAGGTAGGAGCGGGCT-3' |  |
| SIM2-4A-S | 5’-GGGTCGCCGGTGCGTTCGCATATGGAGGTGCTAAGGCCTCCCTTTACAACC-3’ | For SIM2-4A inpGEX-2TK-EBNA1(441-619) |
| SIM2-4A-AS | 5’-GGTTGTAAAGGGAGGCCTTAGCACCTCCATATGCGAACGCACCGGCGACC-3’ |  |
| SSIM3-3A-S | 5’-CCCGCTCCTACCTGCAATGCCAGGGCGACTGCGTGCAGCTTTGACGATGG-3’ | For SIM3-3A in pGEX-2TK-EBNA1(441-619) |
| SIM3-3A-AS | 5’-CCATCGTCAAAGCTGCACGCAGTCGCCCTGGCATTGCAGGTAGGAGCGGG-3’ |  |
| K477R-S | 5'-AGG AGG TTC CAA CCC GAG ATT TGA GAA CAT TGC AG-3' | For K477R mutation of EBNA1 (AJ507799) |
| K477R-AS | 5'-CTG CAA TGT TCT CAA ATC TCG GGT TGG AAC CTC CT-3' |  |
| EBNA1-C-S | 5’-TCGGGATCCGACCCAGGAGAAGGCCCAA-3’ | For wild type GST-EBNA1 (441-619 and its mutants construction with *Bam*HI and *Eco*RI |
| EBNA1-C-AS | 5’-GGAATTCCGCGGCAGCCCCTTCCACCATA-3’ |  |
| Orip-DS F | 5’-CTG CCC TTG TGA CTA AAA TGG TTC-3’ | For ChIP to detect EBNA1 DNA-binding ability |
| Orip-DS R | 5’-AGC GGG TGT TGG CGG GTG TC-3’ |  |
