# Supplementary material for: STUB1 is targeted by the SUMO-interacting motif of EBNA1 to maintain Epstein-Barr Virus latency
Source: PLoS Pathog. 2020 Mar 16;16(3):e1008447. doi: 10.1371/journal.ppat.1008447 (PMC7105294; doi:10.1371/journal.ppat.1008447)
Supplement: S2 Table — (DOCX) [file ppat.1008447.s002.docx]

**Table S2**. List of SUMO1-associated proteins in the presence of EBNA1 identified by Mass Spectrum analysis

with significantly difference (≥2 fold).

| Protein Name | Descriptions | Vector | EBNA1 |
| --- | --- | --- | --- |
| RPL7 | 60S ribosomal protein L7 | 0.24 | 0.11 |
| KRT2 | Keratin, type II cytoskeletal 2 epidermal | 0.16 | 0 |
| HIST1H1T | Histone H1t | 0.15 | 0 |
| RPS5 | 40S ribosomal protein S5 | 0.15 | 0 |
| EEF1G | Elongation factor 1-gamma | 0.14 | 0.07 |
| RPL8 | 60S ribosomal protein L8 | 0.12 | 0 |
| YWHAE | 14-3-3 protein epsilon | 0.11 | 0 |
| RPS3A | 40S ribosomal protein S3a | 0.11 | 0 |
| CCT8 | T-complex protein 1 subunit theta | 0.11 | 0 |
| RANGAP1 | Ran GTPase-activating protein 1 | 0.11 | 0.05 |
| RPL6 | 60S ribosomal protein L6 | 0.1 | 0 |
| IGHG2 | Ig gamma-2 chain C region | 0.09 | 0 |
| LDHB | L-lactate dehydrogenase B chain | 0.09 | 0 |
| TALDO1 | Transaldolase | 0.09 | 0 |
| HSP90AA1 | Heat shock protein HSP 90-alpha | 0.08 | 0 |
| SLC25A3 | Phosphate carrier protein, mitochondrial | 0.08 | 0 |
| TP53 | Cellular tumor antigen p53 | 0.08 | 0 |
| PA2G4 | Proliferation-associated protein 2G4 | 0.08 | 0 |
| HNRNPF | Heterogeneous nuclear ribonucleoprotein F | 0.07 | 0 |
| NONO | Non-POU domain-containing octamer-binding protein | 0.06 | 0 |
| CCT2 |  | 0.06 | 0 |
| CCT7 | T-complex protein 1 subunit eta | 0.06 | 0 |
| VIM | Vimentin | 0.06 | 0 |
| KRT6A | Keratin, type II cytoskeletal 6A | 0.05 | 0 |
| RIMBP2 | RIMS-binding protein 2 | 0.03 | 0 |
| SND1 | Staphylococcal nuclease domain-containing protein 1 | 0.03 | 0 |
| DHX9 | ATP-dependent RNA helicase A | 0.02 | 0 |
| SF3B1 | Splicing factor 3B subunit 1 | 0.02 | 0 |
| SUMO1 | Small ubiquitin-related modifier 1 | 0.3 | 3.79 |
| ACTB | Actin, cytoplasmic 1 | 0.16 | 1.14 |
| HSPA5 | 78 kDa glucose-regulated protein | 0 | 0.86 |
| KRT10 | Keratin, type I cytoskeletal 10 | 0.11 | 0.82 |
| KRT1 | Keratin, type II cytoskeletal 1 | 0.27 | 0.79 |
| HSPA9 | Stress-70 protein, mitochondrial | 0.14 | 0.76 |
| TUBB4A | Tubulin beta-4A chain | 0 | 0.67 |
| HIST1H2AB | Histone H2A type 1-B/E | 0 | 0.54 |
| TUBA1A | Tubulin alpha-1A chain | 0 | 0.47 |
| RPS16 | 40S ribosomal protein S16 | 0 | 0.45 |
| SLC25A5 | ADP/ATP translocase 2 | 0 | 0.33 |
| RPLP0 | 60S acidic ribosomal protein P0 | 0 | 0.32 |
| SUMO2 | Small ubiquitin-related modifier 2 | 0 | 0.32 |
| HSP90AB1 | Heat shock protein HSP 90-beta | 0 | 0.31 |
|  | Ig kappa chain V-II region Cum | 0 | 0.27 |
| ATP5B | ATP synthase subunit beta, mitochondrial | 0 | 0.25 |
| HIST1H2BB | Histone H2B type 1-B | 0 | 0.24 |
| HNRNPH1 | Heterogeneous nuclear ribonucleoprotein H | 0 | 0.21 |
| HNRNPC | Heterogeneous nuclear ribonucleoproteins C1/C2 | 0 | 0.21 |
| HNRNPK | Heterogeneous nuclear ribonucleoprotein K | 0 | 0.21 |
| RPSA | 40S ribosomal protein SA | 0 | 0.21 |
| RPL27A | 60S ribosomal protein L27a | 0 | 0.2 |
| RPL12 | 60S ribosomal protein L12 | 0 | 0.19 |
| RPL24 | 60S ribosomal protein L24 | 0 | 0.19 |
| RPS18 | 40S ribosomal protein S18 | 0 | 0.19 |
| RPL11 | 60S ribosomal protein L11 | 0 | 0.17 |
| RPL18 | 60S ribosomal protein L18 | 0 | 0.16 |
| HSPB1 | Heat shock protein beta-1 | 0 | 0.15 |
| NDUFB9 | NADH dehydrogenase [ubiquinone] 1 beta subcomplex subunit 9 | 0 | 0.15 |
| RPS9 | 40S ribosomal protein S9 | 0 | 0.15 |
| EEF1A1 | Elongation factor 1-alpha 1 | 0 | 0.14 |
| ENO1 | Alpha-enolase | 0 | 0.14 |
| GSTP1 | Glutathione S-transferase P | 0 | 0.14 |
| PPIB | Peptidyl-prolyl cis-trans isomerase B | 0 | 0.14 |
| RPS8 | 40S ribosomal protein S8 | 0 | 0.14 |
| DDX17 | Probable ATP-dependent RNA helicase DDX17 | 0 | 0.13 |
| RPS3 | 40S ribosomal protein S3 | 0 | 0.13 |
| TMEM109 | Transmembrane protein 109 | 0 | 0.13 |
| PKM | Pyruvate kinase PKM | 0 | 0.12 |
| PRDX3 | Thioredoxin-dependent peroxide reductase, mitochondrial | 0 | 0.12 |
| DPM1 | Dolichol-phosphate mannosyltransferase subunit 1 | 0 | 0.11 |
| ECHS1 | Enoyl-CoA hydratase, mitochondrial | 0 | 0.11 |
| PHB | Prohibitin | 0 | 0.11 |
| RPL7 | 60S ribosomal protein L7 | 0 | 0.11 |
| RPL7A | 60S ribosomal protein L7a | 0 | 0.11 |
| RPS2 | 40S ribosomal protein S2 | 0 | 0.11 |
| RPS4X | 40S ribosomal protein S4, X isoform | 0 | 0.11 |
| CCT3 | T-complex protein 1 subunit gamma | 0 | 0.11 |
| CCT8 | T-complex protein 1 subunit theta | 0 | 0.11 |
| LRRC59 | Leucine-rich repeat-containing protein 59 | 0 | 0.1 |
| HNRNPD | Heterogeneous nuclear ribonucleoprotein D0 | 0 | 0.09 |
| HNRNPM | Heterogeneous nuclear ribonucleoprotein M | 0 | 0.09 |
| MDH2 | Malate dehydrogenase, mitochondrial | 0 | 0.09 |
| PCBP2 | Poly(rC)-binding protein 2 | 0 | 0.09 |
| PPP1CC | Serine/threonine-protein phosphatase PP1-gamma catalytic subunit | 0 | 0.09 |
| ILF2 | Interleukin enhancer-binding factor 2 | 0 | 0.08 |
| PDHB | Pyruvate dehydrogenase E1 component subunit beta, mitochondrial | 0 | 0.08 |
| DDX39A | ATP-dependent RNA helicase DDX39A | 0 | 0.07 |
| TUFM | Elongation factor Tu, mitochondrial | 0 | 0.07 |
| EIF4A1 | Eukaryotic initiation factor 4A-I | 0 | 0.07 |
| PDIA6 | Protein disulfide-isomerase A6 | 0 | 0.07 |
| AHCY | Adenosylhomocysteinase | 0 | 0.07 |
| G3BP1 | Ras GTPase-activating protein-binding protein 1 | 0 | 0.06 |
| DDOST | Dolichyl-diphosphooligosaccharide--protein glycosyltransferase 48 kDa subunit | 0 | 0.06 |
| CCT6A | T-complex protein 1 subunit zeta | 0 | 0.06 |
| CANX | Calnexin | 0 | 0.05 |
| EZR | Ezrin | 0 | 0.05 |
| PDIA4 | Protein disulfide-isomerase A4 | 0 | 0.05 |
| TKT | Transketolase | 0 | 0.05 |
| RANBP2 | E3 SUMO-protein ligase RanBP2 | 0.02 | 0.04 |
| MCM3 | DNA replication licensing factor MCM3 | 0 | 0.04 |
| NCL | Nucleolin | 0 | 0.04 |
| ALDH18A1 | Delta-1-pyrroline-5-carboxylate synthase | 0 | 0.04 |
| SSRP1 | FACT complex subunit SSRP1 | 0 | 0.04 |
| TARS | Threonine--tRNA ligase, cytoplasmic | 0 | 0.04 |
| TRIM28 | Transcription intermediary factor 1-beta | 0 | 0.04 |
| MTHFD1 | C-1-tetrahydrofolate synthase, cytoplasmic | 0 | 0.03 |
| GTF2I | General transcription factor II-I | 0 | 0.03 |
| KPNB1 | Importin subunit beta-1 | 0 | 0.03 |
| MATR3 | Matrin-3 | 0 | 0.03 |
| UBA1 | Ubiquitin-like modifier-activating enzyme 1 | 0 | 0.03 |
| ABCC9 | ATP-binding cassette sub-family C member 9 | 0 | 0.02 |
| CLTC | Clathrin heavy chain 1 | 0 | 0.02 |
| CAD | CAD protein | 0 | 0.01 |
