# Supplementary material for: STUB1 is targeted by the SUMO-interacting motif of EBNA1 to maintain Epstein-Barr Virus latency
Source: PLoS Pathog. 2020 Mar 16;16(3):e1008447. doi: 10.1371/journal.ppat.1008447 (PMC7105294; doi:10.1371/journal.ppat.1008447)
Supplement: S3 Table — (DOCX) [file ppat.1008447.s003.docx]

**Table S3**. List of **SUMO2**-associated proteins in the presence of EBNA1 identified by Mass Spectrum analysis

with significantly difference (≥2 fold).

| Protein Name | Descriptions | Vector | EBNA1 |
| --- | --- | --- | --- |
| CKB | Creatine kinase B-type | 2.02 | 0 |
| HSPE1 | 10 kDa heat shock protein, mitochondrial | 1.68 | 0 |
| TUBB8 | Tubulin beta-8 chain | 1.46 | 0 |
| RPL35 | 60S ribosomal protein L35 | 0.98 | 0 |
| YWHAB | 14-3-3 protein beta/alpha | 0.96 | 0 |
| BANF1 | Barrier-to-autointegration factor | 0.8 | 0 |
| RPL10A | 60S ribosomal protein L10a | 0.67 | 0 |
| SUB1 | Activated RNA polymerase II transcriptional coactivator p15 | 0.65 | 0 |
| TBCA | Tubulin-specific chaperone A | 0.6 | 0 |
| MYL12A | Myosin regulatory light chain 12A | 0.6 | 0 |
| CBX3 | Chromobox protein homolog 3 | 0.57 | 0 |
| YWHAH | 14-3-3 protein eta | 0.56 | 0 |
| MRPL12 | 39S ribosomal protein L12, mitochondrial | 0.55 | 0 |
| HSP90B1 | Endoplasmin | 0.5 | 0 |
| RPS28 | 40S ribosomal protein S28 | 0.45 | 0 |
| ETFB | Electron transfer flavoprotein subunit beta | 0.45 | 0 |
| PFDN5 | Prefoldin subunit 5 | 0.43 | 0 |
| TKT | Transketolase | 0.35 | 0 |
| SF3A1 | Phosphoserine aminotransferase | 0.33 | 0 |
| TPD52L2 | Tumor protein D54 | 0.32 | 0 |
| LSM2 | U6 snRNA-associated Sm-like protein LSm2 | 0.32 | 0 |
| RAB5C | Ras-related protein Rab-5C | 0.31 | 0 |
| SNRPD3 | Small nuclear ribonucleoprotein Sm D3 | 0.3 | 0 |
| RAB14 | Ras-related protein Rab-14 | 0.3 | 0 |
| NDUFS3 | NADH dehydrogenase [ubiquinone] iron-sulfur protein 3, mitochondrial | 0.29 | 0 |
| ERH | Enhancer of rudimentary homolog | 0.28 | 0 |
| MTPN | Myotrophin | 0.27 | 0 |
| EIF6 | Eukaryotic translation initiation factor 6 | 0.27 | 0 |
|  | Ig heavy chain V-III region GA | 0.26 | 0 |
| TPM4 | Tropomyosin alpha-4 chain | 0.25 | 0 |
| PAAF1 | Proteasomal ATPase-associated factor 1 | 0.25 | 0 |
| KRT79 | Keratin, type II cytoskeletal 79 | 0.25 | 0 |
| CLIC4 | Chloride intracellular channel protein 4 | 0.25 | 0 |
| KRT19 | Keratin, type I cytoskeletal 19 | 0.24 | 0 |
| TPTE2P1 | Putative phosphatidylinositol 3,4,5-trisphosphate 3-phosphatase TPTE2P1 | 0.22 | 0 |
| CRABP2 | Cellular retinoic acid-binding protein 2 | 0.22 | 0 |
| GOLT1B | Vesicle transport protein GOT1B | 0.22 | 0 |
| TPM1 | Tropomyosin alpha-1 chain | 0.21 | 0 |
| **UBE2D2** | Ubiquitin-conjugating enzyme E2 D2 | 0.2 | 0 |
| PARK7 | Protein DJ-1 | 0.2 | 0 |
| MAGOHB | Protein mago nashi homolog 2 | 0.2 | 0 |
| KPNA2 | Importin subunit alpha-1 | 0.2 | 0 |
| FIS1 | Mitochondrial fission 1 protein | 0.2 | 0 |
| PRPF19 | Pre-mRNA-processing factor 19 | 0.19 | 0 |
| LAMTOR1 | Ragulator complex protein LAMTOR1 | 0.19 | 0 |
| EIF3M | Eukaryotic translation initiation factor 3 subunit M | 0.19 | 0 |
| CLTCL1 | Clathrin heavy chain 2 | 0.19 | 0 |
| ATP5D | ATP synthase subunit delta, mitochondrial | 0.19 | 0 |
| AASDHPPT | L-aminoadipate-semialdehyde dehydrogenase-phosphopantetheinyl transferase | 0.19 | 0 |
| SNX3 | Sorting nexin-3 | 0.18 | 0 |
| PPAT | Amidophosphoribosyltransferase | 0.18 | 0 |
| SERBP1 | Plasminogen activator inhibitor 1 RNA-binding protein | 0.18 | 0 |
| LDHAL6B | L-lactate dehydrogenase A-like 6B | 0.18 | 0 |
| CUTA | Protein CutA | 0.18 | 0 |
| ATP5H | ATP synthase subunit d, mitochondrial | 0.18 | 0 |
| TPRKB | EKC/KEOPS complex subunit TPRKB | 0.17 | 0 |
| SUGT1 | Suppressor of G2 allele of SKP1 homolog | 0.17 | 0 |
| HDDC2 | HD domain-containing protein 2 | 0.17 | 0 |
| TNFAIP8L1 | Tumor necrosis factor alpha-induced protein 8-like protein 1 | 0.16 | 0 |
| PDHA2 | Pyruvate dehydrogenase E1 component subunit alpha, testis-specific form, mitochondrial | 0.16 | 0 |
| NDUFB10 | NADH dehydrogenase [ubiquinone] 1 beta subcomplex subunit 10 | 0.16 | 0 |
| MANF | Mesencephalic astrocyte-derived neurotrophic factor | 0.16 | 0 |
| GGCT | Gamma-glutamylcyclotransferase | 0.16 | 0 |
| VRK1 | Serine/threonine-protein kinase VRK1 | 0.15 | 0 |
| UBE2K | Ubiquitin-conjugating enzyme E2 K | 0.15 | 0 |
| RHOA | Transforming protein RhoA | 0.15 | 0 |
| PSMB6 | Proteasome subunit beta type-6 | 0.15 | 0 |
| PSMB2 | Proteasome subunit beta type-2 | 0.15 | 0 |
| PAFAH1B2 | Platelet-activating factor acetylhydrolase IB subunit beta | 0.15 | 0 |
| DRAP1 | Dr1-associated corepressor | 0.15 | 0 |
| HEBP2 | Heme-binding protein 2 | 0.15 | 0 |
| CSNK2A3 | Casein kinase II subunit alpha 3 | 0.15 | 0 |
| C16orf80 | UPF0468 protein C16orf80 | 0.15 | 0 |
| TXNDC5 | Thioredoxin domain-containing protein 5 | 0.14 | 0 |
| SCLY | Selenocysteine lyase | 0.14 | 0 |
| PSMD12 | 26S proteasome non-ATPase regulatory subunit 12 | 0.14 | 0 |
| PSMC3 | 26S protease regulatory subunit 6A | 0.14 | 0 |
| NUDT5 | ADP-sugar pyrophosphatase | 0.14 | 0 |
| EIF4H | Eukaryotic translation initiation factor 4H | 0.14 | 0 |
| GLTP | Glycolipid transfer protein | 0.14 | 0 |
| FUS | RNA-binding protein FUS | 0.14 | 0 |
| BYSL | Bystin | 0.14 | 0 |
| LAP3 | Cytosol aminopeptidase | 0.14 | 0 |
| TSN | Translin | 0.13 | 0 |
| TMEM33 | Transmembrane protein 33 | 0.13 | 0 |
| TMEM109 | Transmembrane protein 109 | 0.13 | 0 |
| PSMB5 | Proteasome subunit beta type-5 | 0.13 | 0 |
| PSMA3 | Proteasome subunit alpha type-3 | 0.13 | 0 |
| MOB1A | MOB kinase activator 1A | 0.13 | 0 |
| CLNS1A | Methylosome subunit pICln | 0.13 | 0 |
| GRB2 | Growth factor receptor-bound protein 2 | 0.13 | 0 |
| PROCR | Endothelial protein C receptor | 0.13 | 0 |
| DCXR | L-xylulose reductase | 0.13 | 0 |
| COPS2 | COP9 signalosome complex subunit 2 | 0.13 | 0 |
| ACTN3 | Alpha-actinin-3 | 0.13 | 0 |
| PSME2 | Proteasome activator complex subunit 2 | 0.12 | 0 |
| PSME1 | Proteasome activator complex subunit 1 | 0.12 | 0 |
| PYCRL | Pyrroline-5-carboxylate reductase 3 | 0.12 | 0 |
| HDGF | Hepatoma-derived growth factor | 0.12 | 0 |
| API5 | Apoptosis inhibitor 5 | 0.12 | 0 |
| PUF60 | Poly(U)-binding-splicing factor PUF60 | 0.11 | 0 |
| KATNAL2 | Katanin p60 ATPase-containing subunit A-like 2 | 0.11 | 0 |
| PRKCSH | Glucosidase 2 subunit beta | 0.11 | 0 |
| FUBP3 | Far upstream element-binding protein 3 | 0.11 | 0 |
| EIF3D | Eukaryotic translation initiation factor 3 subunit D | 0.11 | 0 |
| ECHS1 | Enoyl-CoA hydratase, mitochondrial | 0.11 | 0 |
| GLUD1 | Glutamate dehydrogenase 1, mitochondrial | 0.11 | 0 |
| CDK17 | Cyclin-dependent kinase 17 | 0.11 | 0 |
| TOMM34 | Mitochondrial import receptor subunit TOM34 | 0.1 | 0 |
| SSR1 | Translocon-associated protein subunit alpha | 0.1 | 0 |
| MRPL4 | 39S ribosomal protein L4, mitochondrial | 0.1 | 0 |
| NIPSNAP1 | Protein NipSnap homolog 1 | 0.1 | 0 |
| MAK | Serine/threonine-protein kinase MAK | 0.1 | 0 |
| ISOC1 | Isochorismatase domain-containing protein 1 | 0.1 | 0 |
| IER5 | Immediate early response gene 5 protein | 0.1 | 0 |
| CRKL | Crk-like protein | 0.1 | 0 |
| CNN2 | Calponin-2 | 0.1 | 0 |
| BUB3 | Mitotic checkpoint protein BUB3 | 0.1 | 0 |
| AKR1B1 | Aldose reductase | 0.1 | 0 |
| **UCHL5** | Ubiquitin carboxyl-terminal hydrolase isozyme L5 | 0.09 | 0 |
| STYXL1 | Serine/threonine/tyrosine-interacting-like protein 1 | 0.09 | 0 |
| UBA2 | SUMO-activating enzyme subunit 2 | 0.09 | 0 |
| SAE1 | SUMO-activating enzyme subunit 1 | 0.09 | 0 |
| PDXK | Pyridoxal kinase | 0.09 | 0 |
| PDLIM1 | PDZ and LIM domain protein 1 | 0.09 | 0 |
| MAGEF1 | Melanoma-associated antigen F1 | 0.09 | 0 |
| GLRX3 | Glutaredoxin-3 | 0.09 | 0 |
| ST13 | Hsc70-interacting protein | 0.09 | 0 |
| DRG2 | Developmentally-regulated GTP-binding protein 2 | 0.09 | 0 |
| DIMT1 | Probable dimethyladenosine transferase | 0.09 | 0 |
| TMEM236 | Transmembrane protein 236 | 0.08 | 0 |
| TFG | Protein TFG | 0.08 | 0 |
| GTF2A1 | Transcription initiation factor IIA subunit 1 | 0.08 | 0 |
| SYAP1 | Synapse-associated protein 1 | 0.08 | 0 |
| RBMX | RNA-binding motif protein, X chromosome | 0.08 | 0 |
| PPME1 | Protein phosphatase methylesterase 1 | 0.08 | 0 |
| HSPBP1 | Hsp70-binding protein 1 | 0.08 | 0 |
| BRE | BRCA1-A complex subunit BRE | 0.08 | 0 |
| **USP5** | Ubiquitin carboxyl-terminal hydrolase 5 | 0.07 | 0 |
| TTC4 | Tetratricopeptide repeat protein 4 | 0.07 | 0 |
| PLIN3 | Perilipin-3 | 0.07 | 0 |
| PHAX | Phosphorylated adapter RNA export protein | 0.07 | 0 |
| DLST | Dihydrolipoyllysine-residue succinyltransferase component of 2-oxoglutarate dehydrogenase complex, mitochondrial | 0.07 | 0 |
| LAMP1 | Lysosome-associated membrane glycoprotein 1 | 0.07 | 0 |
| CPOX | Oxygen-dependent coproporphyrinogen-III oxidase, mitochondrial | 0.07 | 0 |
| FGFR1 | Fibroblast growth factor receptor 1 | 0.07 | 0 |
| DDX19A | ATP-dependent RNA helicase DDX19A | 0.07 | 0 |
| CTSD | Cathepsin D | 0.07 | 0 |
| GOT1 | Aspartate aminotransferase, cytoplasmic | 0.07 | 0 |
| XPNPEP3 | Probable Xaa-Pro aminopeptidase 3 | 0.06 | 0 |
| **USP14** | Ubiquitin carboxyl-terminal hydrolase 14 | 0.06 | 0 |
| **U2AF2** | Splicing factor U2AF 65 kDa subunit | 0.06 | 0 |
| TRIM35 | Tripartite motif-containing protein 35 | 0.06 | 0 |
| ALDH5A1 | Succinate-semialdehyde dehydrogenase, mitochondrial | 0.06 | 0 |
| SKIV2L2 | Superkiller viralicidic activity 2-like 2 | 0.06 | 0 |
| SAAL1 | Protein SAAL1 | 0.06 | 0 |
| SNRNP70 | U1 small nuclear ribonucleoprotein 70 kDa | 0.06 | 0 |
| PWP1 | Periodic tryptophan protein 1 homolog | 0.06 | 0 |
| NMT1 | Glycylpeptide N-tetradecanoyltransferase 1 | 0.06 | 0 |
| MYH11 | Myosin-11 | 0.06 | 0 |
| MORC4 | MORC family CW-type zinc finger protein 4 | 0.06 | 0 |
| DNAJC7 | DnaJ homolog subfamily C member 7 | 0.06 | 0 |
| DHCR7 | 7-dehydrocholesterol reductase | 0.06 | 0 |
| CYP51A1 | Lanosterol 14-alpha demethylase | 0.06 | 0 |
| MTHFD1L | Monofunctional C1-tetrahydrofolate synthase, mitochondrial | 0.06 | 0 |
| BLMH | Bleomycin hydrolase | 0.06 | 0 |
| ATP2A2 | Sarcoplasmic/endoplasmic reticulum calcium ATPase 2 | 0.06 | 0 |
| MAOA | Amine oxidase [flavin-containing] A | 0.06 | 0 |
| ALDH7A1 | Alpha-aminoadipic semialdehyde dehydrogenase | 0.06 | 0 |
| WDR72 | WD repeat-containing protein 72 | 0.05 | 0 |
| RGPD1 | RANBP2-like and GRIP domain-containing protein 1 | 0.05 | 0 |
| MTPAP | Poly(A) RNA polymerase, mitochondrial | 0.05 | 0 |
| MAN2A1 | Alpha-mannosidase 2 | 0.05 | 0 |
| KBTBD3 | Kelch repeat and BTB domain-containing protein 3 | 0.05 | 0 |
| ISYNA1 | Inositol-3-phosphate synthase 1 | 0.05 | 0 |
| GLMN | Glomulin | 0.05 | 0 |
| SKIV2L2 | Splicing factor 3A subunit 1 | 0.04 | 0 |
| GAPVD1 | GTPase-activating protein and VPS9 domain-containing protein 1 | 0.04 | 0 |
| CAPRIN1 | Caprin-1 | 0.04 | 0 |
| TDRD5 | Tudor domain-containing protein 5 | 0.03 | 0 |
| SEL1L3 | Protein sel-1 homolog 3 | 0.03 | 0 |
| PRPF40A | Pre-mRNA-processing factor 40 homolog A | 0.03 | 0 |
| OSBPL8 | Oxysterol-binding protein-related protein 8 | 0.03 | 0 |
| NAA25 | N-alpha-acetyltransferase 25, NatB auxiliary subunit | 0.03 | 0 |
| KIF11 | Kinesin-like protein KIF11 | 0.03 | 0 |
| KIAA1377 | Uncharacterized protein KIAA1377 | 0.03 | 0 |
| IQCA1P1 | Putative IQ and AAA domain-containing protein 1-like | 0.03 | 0 |
| IPO8 | Importin-8 OS=Homo sapiens GN=IPO8 PE=1 SV=2 | 0.03 | 0 |
| EIF4G2 | Eukaryotic translation initiation factor 4 gamma 2 | 0.03 | 0 |
| HK1 | Hexokinase-1 | 0.03 | 0 |
| FLNC | Filamin-C | 0.03 | 0 |
| ECM29 | Proteasome-associated protein ECM29 homolog | 0.03 | 0 |
| CYFIP1 | Cytoplasmic FMR1-interacting protein 1 | 0.03 | 0 |
| CHD4 | Chromodomain-helicase-DNA-binding protein 4 | 0.03 | 0 |
| BCLAF1 | Bcl-2-associated transcription factor 1 | 0.03 | 0 |
| ATP2A3 | Sarcoplasmic/endoplasmic reticulum calcium ATPase 3 | 0.03 | 0 |
| ANKRD26 | Ankyrin repeat domain-containing protein 26 | 0.03 | 0 |
| UBE2O | E2/E3 hybrid ubiquitin-protein ligase UBE2O | 0.02 | 0 |
| SMC1B | Structural maintenance of chromosomes protein 1B | 0.02 | 0 |
| RTL1 | Retrotransposon-like protein 1 | 0.02 | 0 |
| GRIN2C | Glutamate receptor ionotropic, NMDA 2C | 0.02 | 0 |
| EVC2 | Limbin | 0.02 | 0 |
| HRNR | Hornerin | 0.02 | 0 |
| FLII | Protein flightless-1 homolog | 0.02 | 0 |
| DNMT1 | DNA (cytosine-5)-methyltransferase 1 | 0.02 | 0 |
| CLASP1 | CLIP-associating protein 1 | 0.02 | 0 |
| FER1L5 | Fer-1-like protein 5 | 0.01 | 0 |
| CSTB | Cystatin-B | 1.95 | 0.31 |
| VDAC2 | Voltage-dependent anion-selective channel protein 2 | 0.54 | 0.11 |
| HSD17B10 | 3-hydroxyacyl-CoA dehydrogenase type-2 | 0.85 | 0.18 |
| VCL | Vinculin | 0.11 | 0.03 |
| CA2 | Carbonic anhydrase 2 | 0.48 | 0.14 |
| PEBP1 | Phosphatidylethanolamine-binding protein 1 | 0.75 | 0.22 |
| TOMM40 | Mitochondrial import receptor subunit TOM40 homolog | 0.29 | 0.09 |
| CALR | Calreticulin | 0.22 | 0.07 |
| PRDX1 | Peroxiredoxin-1 | 2.76 | 0.88 |
| RAD23B | UV excision repair protein RAD23 homolog B | 0.25 | 0.08 |
| TAGLN2 | Transgelin-2 | 1.53 | 0.49 |
| EPRS | Bifunctional glutamate/proline--tRNA ligase | 0.06 | 0.02 |
| EIF4G1 | Eukaryotic translation initiation factor 4 gamma 1 | 0.06 | 0.02 |
| TPI1 | Triosephosphate isomerase | 1.24 | 0.42 |
| CS | Citrate synthase, mitochondrial | 0.44 | 0.16 |
| NME1 | Nucleoside diphosphate kinase A | 6.28 | 2.54 |
| RPS21 | 40S ribosomal protein S21 | 0.91 | 0.38 |
| NEDD8 | NEDD8 | 0.91 | 0.38 |
| NME2 | Nucleoside diphosphate kinase B | 6.06 | 2.54 |
| GSTP1 | Glutathione S-transferase P | 1.06 | 0.46 |
| RAB7A | Ras-related protein Rab-7a | 0.71 | 0.31 |
| TXN | Thioredoxin | 0.87 | 0.38 |
| CDKN2A | Cyclin-dependent kinase inhibitor 2A, isoforms 1/2/3 | 0.45 | 0.2 |
| ATIC | Bifunctional purine biosynthesis protein PURH | 0.51 | 0.23 |
| GSTM2 | Glutathione S-transferase Mu 2 | 0.28 | 0.13 |
| FKBP3 | Peptidyl-prolyl cis-trans isomerase FKBP3 | 0.28 | 0.13 |
| ERP29 | Endoplasmic reticulum resident protein 29 | 0.28 | 0.13 |
| UBE2N | Ubiquitin-conjugating enzyme E2 N | 0.43 | 0.2 |
| BZW2 | Basic leucine zipper and W2 domain-containing protein 2 | 0.3 | 0.14 |
| LAMP2 | Lysosome-associated membrane glycoprotein 2 | 0.15 | 0.07 |
| RPN2 | Dolichyl-diphosphooligosaccharide--protein glycosyltransferase subunit 2 | 0.32 | 0.15 |
| PSMA5 | Proteasome subunit alpha type-5 | 0.34 | 0.16 |
| PSMD13 | 26S proteasome non-ATPase regulatory subunit 13 | 0.17 | 0.08 |
| PRDX5 | Peroxiredoxin-5, mitochondrial | 0.38 | 0.18 |
| HYOU1 | Hypoxia up-regulated protein 1 | 0.19 | 0.09 |
| VDAC3 | Voltage-dependent anion-selective channel protein 3 | 0.25 | 0.12 |
| AK2 | Adenylate kinase 2, mitochondrial | 0.27 | 0.13 |
| DUT | Deoxyuridine 5~-triphosphate nucleotidohydrolase, mitochondrial | 0.27 | 0.13 |
| MTAP | S-methyl-5~-thioadenosine phosphorylase | 0.22 | 0.11 |
| FSCN1 | Fascin | 0.12 | 0.06 |
| DLAT | Dihydrolipoyllysine-residue acetyltransferase component of pyruvate dehydrogenase complex, mitochondrial | 0.1 | 0.05 |
| FLNB | Filamin-B | 0.04 | 0.02 |
| ALDOA | Fructose-bisphosphate aldolase A | 0.62 | 1.23 |
| STUB1 | E3 ubiquitin-protein ligase CHIP | 0.1 | 0.2 |
| PSMD6 | 26S proteasome non-ATPase regulatory subunit 6 | 0.08 | 0.16 |
| FH | Fumarate hydratase, mitochondrial | 0.06 | 0.12 |
| SETX | Probable helicase senataxin | 0.01 | 0.02 |
| SNRPA1 | U2 small nuclear ribonucleoprotein A | 0.13 | 0.27 |
| MAPRE1 | Microtubule-associated protein RP/EB family member 1 | 0.11 | 0.23 |
| RAP1A | Ras-related protein Rap-1A | 0.19 | 0.4 |
| PSMA2 | Proteasome subunit alpha type-2 | 0.17 | 0.36 |
| EEF1B2 | Elongation factor 1-beta | 0.15 | 0.32 |
| MDH1 | Malate dehydrogenase, cytoplasmic | 0.21 | 0.46 |
| TBC1D32 | Protein broad-minded | 0.02 | 0.05 |
| FARSA | Phenylalanine--tRNA ligase alpha subunit | 0.06 | 0.19 |
| RANGAP1 | Ran GTPase-activating protein 1 | 0.11 | 0.37 |
| PDCD6IP | Programmed cell death 6-interacting protein | 0.03 | 0.11 |
| EZR | Ezrin | 0.05 | 0.21 |
| FARSB | Phenylalanine--tRNA ligase beta subunit O | 0.05 | 0.22 |
| XPO1 | Exportin-1 | 0.03 | 0.19 |
| RPL23A | 60S ribosomal protein L23a | 0 | 3.36 |
| RPL38 | 60S ribosomal protein L38 | 0 | 3.02 |
| TUBA1B | Tubulin alpha-1B chain | 0 | 2.57 |
| LDHA | L-lactate dehydrogenase A chain | 0 | 1.53 |
| RPL15 | 60S ribosomal protein L15 | 0 | 1.3 |
| RPL26L1 | 60S ribosomal protein L26-like 1 | 0 | 1.25 |
| RPS19 | 40S ribosomal protein S19 | 0 | 0.83 |
| RPL18 | 60S ribosomal protein L18 | 0 | 0.78 |
| PSMD11 | 26S proteasome non-ATPase regulatory subunit 11 | 0 | 0.7 |
| HSPA1L | Heat shock 70 kDa protein 1-like | 0 | 0.56 |
| RBM3 | Putative RNA-binding protein 3 | 0 | 0.51 |
| CPLX1 | Complexin-1 | 0 | 0.51 |
| KRT15 | Keratin, type I cytoskeletal 15 | 0 | 0.48 |
| SET | Protein SET | 0 | 0.46 |
| DDX39B | Spliceosome RNA helicase DDX39B | 0 | 0.41 |
| KRT13 | Keratin, type I cytoskeletal 13 | 0 | 0.38 |
| ATP5C1 | ATP synthase subunit gamma, mitochondrial | 0 | 0.37 |
|  | Ig kappa chain V-II region Cum | 0 | 0.34 |
| KRT76 | Keratin, type II cytoskeletal 2 oral | 0 | 0.28 |
| TCEB2 | Transcription elongation factor B polypeptide 2 | 0 | 0.26 |
| CENPH | Centromere protein H | 0 | 0.26 |
| NDUFA5 | NADH dehydrogenase [ubiquinone] 1 alpha subcomplex subunit 5 | 0 | 0.25 |
| DDTL | D-dopachrome decarboxylase-like protein | 0 | 0.24 |
| ATP5F1 | ATP synthase F(0) complex subunit B1, mitochondrial | 0 | 0.24 |
| DHX9 | ATP-dependent RNA helicase A | 0 | 0.23 |
| TSHB | Thyrotropin subunit beta | 0 | 0.22 |
| TACO1 | Translational activator of cytochrome c oxidase 1 | 0 | 0.22 |
| PCBP1 | Poly(rC)-binding protein 1 | 0 | 0.22 |
| SRSF3 | Serine/arginine-rich splicing factor 3 | 0 | 0.21 |
| CDK5 | Cyclin-dependent kinase 5 | 0 | 0.21 |
| PDHB | Pyruvate dehydrogenase E1 component subunit beta, mitochondrial | 0 | 0.2 |
| HNRNPA3 | Heterogeneous nuclear ribonucleoprotein A3 | 0 | 0.19 |
| MRPS23 | 28S ribosomal protein S23, mitochondrial | 0 | 0.18 |
| MRPL49 | 39S ribosomal protein L49, mitochondrial | 0 | 0.18 |
| RCC1 | Regulator of chromosome condensation | 0 | 0.18 |
| KRT74 | Keratin, type II cytoskeletal 74 | 0 | 0.18 |
| SORD | Sorbitol dehydrogenase | 0 | 0.18 |
| CANX | Calnexin | 0 | 0.18 |
| PRPSAP2 | Phosphoribosyl pyrophosphate synthase-associated protein 2 | 0 | 0.17 |
| FEN1 | Flap endonuclease 1 | 0 | 0.17 |
| DNAJC25 | DnaJ homolog subfamily C member 25 | 0 | 0.17 |
| CBX1 | Chromobox protein homolog 1 | 0 | 0.16 |
| ARL8A | ADP-ribosylation factor-like protein 8A | 0 | 0.16 |
| TMED2 | Transmembrane emp24 domain-containing protein 2 | 0 | 0.15 |
| SAR1A | GTP-binding protein SAR1a | 0 | 0.15 |
| MRPL21 | 39S ribosomal protein L21, mitochondrial | 0 | 0.15 |
| OSTF1 | Osteoclast-stimulating factor 1 | 0 | 0.14 |
| ATP6V1E1 | V-type proton ATPase subunit E 1 | 0 | 0.14 |
| CHCHD3 | Coiled-coil-helix-coiled-coil-helix domain-containing protein 3, mitochondrial | 0 | 0.13 |
| SNTB2 | Beta-2-syntrophin | 0 | 0.12 |
| SLC25A13 | Chloride intracellular channel protein 2 | 0 | 0.12 |
| SNRPA | U1 small nuclear ribonucleoprotein A | 0 | 0.11 |
| PPP2CA | Serine/threonine-protein phosphatase 2A catalytic subunit alpha isoform | 0 | 0.11 |
| CCDC28A | Coiled-coil domain-containing protein 28A | 0 | 0.11 |
| CAPZB | F-actin-capping protein subunit beta | 0 | 0.11 |
| APEX1 | DNA-(apurinic or apyrimidinic site) lyase | 0 | 0.11 |
| TXNL1 | Thioredoxin-like protein 1 | 0 | 0.1 |
| SGTA | Small glutamine-rich tetratricopeptide repeat-containing protein alpha | 0 | 0.1 |
| SF1 | Splicing factor 1 | 0 | 0.1 |
| PYGM | Glycogen phosphorylase, muscle form | 0 | 0.1 |
| PSMD14 | 26S proteasome non-ATPase regulatory subunit 14 | 0 | 0.1 |
| PGP | Phosphoglycolate phosphatase | 0 | 0.1 |
| MRI1 | Methylthioribose-1-phosphate isomerase | 0 | 0.1 |
| GIPC2 | PDZ domain-containing protein GIPC2 | 0 | 0.1 |
| HSD17B11 | Estradiol 17-beta-dehydrogenase 11 | 0 | 0.1 |
| C2orf47 | Uncharacterized protein C2orf47, mitochondrial | 0 | 0.1 |
| UBXN1 | UBX domain-containing protein 1 | 0 | 0.1 |
| TCEA1 | Transcription elongation factor A protein 1 | 0 | 0.1 |
| GIPC1 | PDZ domain-containing protein GIPC1 | 0 | 0.09 |
| GNB2 | Guanine nucleotide-binding protein G(I)/G(S)/G(T) subunit beta-2 | 0 | 0.09 |
| EIF3H | Eukaryotic translation initiation factor 3 subunit H | 0 | 0.09 |
| SEPHS1 | Selenide, water dikinase 1 | 0 | 0.08 |
| SEC63 | Translocation protein SEC63 homolog | 0 | 0.08 |
| PDHA1 | Pyruvate dehydrogenase E1 component subunit alpha, somatic form, mitochondrial | 0 | 0.08 |
| NSF | Vesicle-fusing ATPase | 0 | 0.08 |
| LUC7L | Putative RNA-binding protein Luc7-like 1 | 0 | 0.08 |
| LPGAT1 | Acyl-CoA:lysophosphatidylglycerol acyltransferase 1 | 0 | 0.08 |
| PRKAR1A | cAMP-dependent protein kinase type I-alpha regulatory subunit | 0 | 0.08 |
| POLD1 | DNA polymerase delta catalytic subunit | 0 | 0.08 |
| ARPC1A | Actin-related protein 2/3 complex subunit 1A | 0 | 0.08 |
| VAC14 | Protein VAC14 homolog | 0 | 0.08 |
| ZC3H15 | Zinc finger CCCH domain-containing protein 15 | 0 | 0.07 |
| RBM17 | Splicing factor 45 | 0 | 0.07 |
| RPL3L | 60S ribosomal protein L3-like | 0 | 0.07 |
| PRIM1 | DNA primase small subunit | 0 | 0.07 |
| ORC4 | Origin recognition complex subunit 4 | 0 | 0.07 |
| NFATC2IP | NFATC2-interacting protein | 0 | 0.07 |
| CKMT1A | Creatine kinase U-type, mitochondrial | 0 | 0.07 |
| HAT1 | Histone acetyltransferase type B catalytic subunit | 0 | 0.07 |
| DNM3 | Dynamin-3 | 0 | 0.07 |
| COPB2 | Coatomer subunit beta | 0 | 0.07 |
| CHAMP1 | Chromosome alignment-maintaining phosphoprotein 1 | 0 | 0.07 |
| ACADM | Medium-chain specific acyl-CoA dehydrogenase, mitochondrial | 0 | 0.07 |
| ZBTB2 | Zinc finger and BTB domain-containing protein 2 | 0 | 0.06 |
| EFTUD2 | 116 kDa U5 small nuclear ribonucleoprotein component | 0 | 0.06 |
| NUP62 | Nuclear pore glycoprotein p62 | 0 | 0.06 |
| NAMPT | Nicotinamide phosphoribosyltransferase | 0 | 0.06 |
| IDE | Insulin-degrading enzyme | 0 | 0.06 |
| GPS1 | COP9 signalosome complex subunit 1 | 0 | 0.06 |
| CRISPLD2 | Cysteine-rich secretory protein LCCL domain-containing 2 | 0 | 0.06 |
| BLK | Tyrosine-protein kinase Blk | 0 | 0.06 |
| SRP68 | Signal recognition particle subunit SRP68 | 0 | 0.05 |
| SP140L | Nuclear body protein SP140-like protein | 0 | 0.05 |
| KEAP1 | Kelch-like ECH-associated protein 1 | 0 | 0.05 |
| FANCI | Fanconi anemia group I protein | 0 | 0.05 |
| EIF2A | Eukaryotic translation initiation factor 2A | 0 | 0.05 |
| DIAPH1 | Protein diaphanous homolog 1 | 0 | 0.05 |
| CSTF2 | Cleavage stimulation factor subunit 2 | 0 | 0.05 |
| CARM1 | Histone-arginine methyltransferase CARM1 | 0 | 0.05 |
| PGM3 | Phosphoacetylglucosamine mutase | 0 | 0.05 |
| ZNF782 | Zinc finger protein 782 | 0 | 0.04 |
| ZNF304 | Zinc finger protein 304 | 0 | 0.04 |
| USP10 | Ubiquitin carboxyl-terminal hydrolase 10 | 0 | 0.04 |
| QARS | Glutamine--tRNA ligase | 0 | 0.04 |
| STON2 | Stonin-2 | 0 | 0.04 |
| SRP72 | Signal recognition particle subunit SRP72 | 0 | 0.04 |
| RASAL1 | RasGAP-activating-like protein 1 | 0 | 0.04 |
| PCDHB6 | Protocadherin beta-6 | 0 | 0.04 |
| PAPOLA | Poly(A) polymerase alpha | 0 | 0.04 |
| LEPRE1 | Prolyl 3-hydroxylase 1 | 0 | 0.04 |
| OSBPL11 | Oxysterol-binding protein-related protein 11 | 0 | 0.04 |
| NOP2 | Putative ribosomal RNA methyltransferase NOP2 | 0 | 0.04 |
| NDUFS1 | NADH-ubiquinone oxidoreductase 75 kDa subunit, mitochondrial | 0 | 0.04 |
| NCBP1 | Nuclear cap-binding protein subunit 1 | 0 | 0.04 |
| KCND3 | Potassium voltage-gated channel subfamily D member 3 | 0 | 0.04 |
| GIT1 | ARF GTPase-activating protein GIT1 | 0 | 0.04 |
| CUL1 | Cullin-1 | 0 | 0.04 |
| CSDE1 | Cold shock domain-containing protein E1 | 0 | 0.04 |
| CROCC | Rootletin | 0 | 0.04 |
| SLC25A13 | Calcium-binding mitochondrial carrier protein Aralar2 | 0 | 0.04 |
| VPS35 | Vacuolar protein sorting-associated protein 35 | 0 | 0.04 |
| TICAM1 | TIR domain-containing adapter molecule 1 | 0 | 0.04 |
| TPR | Nucleoprotein TPR | 0 | 0.03 |
| TNPO3 | Transportin-3 | 0 | 0.03 |
| TMTC3 | Transmembrane and TPR repeat-containing protein 3 | 0 | 0.03 |
| TMC2 | Transmembrane channel-like protein 2 | 0 | 0.03 |
| IARS2 | Isoleucine--tRNA ligase, mitochondrial | 0 | 0.03 |
| MYO1D | Unconventional myosin-Id | 0 | 0.03 |
| LONP1 | Lon protease homolog, mitochondrial | 0 | 0.03 |
| DDX23 | Probable ATP-dependent RNA helicase DDX23 | 0 | 0.03 |
| CHD5 | Chromodomain-helicase-DNA-binding protein 5 | 0 | 0.03 |
| CCAR2 | Cell cycle and apoptosis regulator protein 2 | 0 | 0.03 |
| BRWD3 | Bromodomain and WD repeat-containing protein 3 | 0 | 0.03 |
| BAG6 | Large proline-rich protein BAG6 | 0 | 0.03 |
| AP2A | AP-2 complex subunit alpha-1 | 0 | 0.03 |
| AP1B1 | AP-1 complex subunit beta-1 | 0 | 0.03 |
| TJP2 | Tight junction protein ZO-2 | 0 | 0.02 |
| TLN2 | Talin-2 | 0 | 0.02 |
| PITPNM2 | Membrane-associated phosphatidylinositol transfer protein 2 | 0 | 0.02 |
| NUP188 | Nucleoporin NUP188 homolog | 0 | 0.02 |
| DZIP3 | E3 ubiquitin-protein ligase DZIP3 | 0 | 0.02 |
| CPSF1 | Cleavage and polyadenylation specificity factor subunit 1 | 0 | 0.02 |
| CAMTA1 | Calmodulin-binding transcription activator 1 | 0 | 0.02 |
| CHD3 | Chromodomain-helicase-DNA-binding protein 3 | 0 | 0.02 |
| VPS13B | Vacuolar protein sorting-associated protein 13B | 0 | 0.02 |
| UBR2 | E3 ubiquitin-protein ligase UBR2 | 0 | 0.02 |
| CENPF | Centromere protein F | 0 | 0.01 |
