# Supplementary material for: STUB1 is targeted by the SUMO-interacting motif of EBNA1 to maintain Epstein-Barr Virus latency
Source: PLoS Pathog. 2020 Mar 16;16(3):e1008447. doi: 10.1371/journal.ppat.1008447 (PMC7105294; doi:10.1371/journal.ppat.1008447)
Supplement: S4 Table — (DOCX) [file ppat.1008447.s004.docx]

**Table S4**. List of EBNA1-associated proteins identified by Mass Spectrum analysis with significantly difference (≥2 fold).

| Protein Name | Descriptions | Full Length | ΔSIM2 | ΔSIM3 | ΔSIM2+3 |
| --- | --- | --- | --- | --- | --- |
| RPS6 | 40S ribosomal protein S6 | 0.39 | 0.93 | 0.73 | 0.93 |
| ACAT2 | Acetyl-CoA acetyltransferase, cytosolic |  | 0.08 |  | 0.08 |
| PGD | 6-phosphogluconate dehydrogenase, decarboxylating | 0.13 | 0.2 | 0.27 | 0.27 |
| AGPS | Alkyldihydroxyacetonephosphate synthase, peroxisomal |  | 0.05 |  | 0.09 |
| ADD1 | Alpha-adducin |  | 0.04 | 0.04 | 0.04 |
| AIFM1 | Apoptosis-inducing factor 1, mitochondrial | 0.05 | 0.1 | 0.05 | 0.1 |
| ARPC2 | Actin-related protein 2/3 complex subunit 2 |  | 0.1 | 0.1 | 0.1 |
| ATP5F1 | ATP synthase F(0) complex subunit B1, mitochondrial |  | 0.12 |  | 0.12 |
| BCCIP | BRCA2 and CDKN1A-interacting protein |  | 0.08 | 0.09 | 0.09 |
| CAPRIN1 | Caprin-1 |  |  | 0.04 | 0.04 |
| CLUH | Clustered mitochondria protein homolog | 0.02 | 0.04 |  | 0.09 |
| NCAPD2 | Condensin complex subunit 1 | 0.02 | 0.02 | 0.04 | 0.04 |
| CTNNBL1 | Beta-catenin-like protein 1 |  | 0.05 | 0.05 | 0.05 |
| CUL1 | Cullin-1 |  | 0.04 | 0.04 | 0.04 |
| CUL4A | Cullin-4A | 0.04 |  | 0.12 | 0.08 |
| SPECC1 | Cytospin-B |  |  | 0.03 | 0.03 |
| DHCR7 | 7-dehydrocholesterol reductase |  | 0.06 | 0.06 | 0.06 |
| HADHB | Trifunctional enzyme subunit beta, mitochondrial |  |  | 0.13 | 0.13 |
| EFR3A | Protein EFR3 homolog A |  |  | 0.04 | 0.04 |
| EIF3C | Eukaryotic translation initiation factor 3 subunit C |  | 0.13 |  | 0.13 |
| EIF3E | Eukaryotic translation initiation factor 3 subunit E |  | 0.06 | 0.06 | 0.06 |
| EIF3M | Eukaryotic translation initiation factor 3 subunit M |  | 0.08 | 0.08 | 0.16 |
| FAF2 | FAS-associated factor 2 |  | 0.06 | 0.13 | 0.2 |
| FBXO46 | F-box only protein 46 |  | 0.05 | 0.05 | 0.05 |
| FLNA | Filamin-A |  | 0.05 | 0.04 | 0.02 |
| H1FX | Histone H1x |  | 0.15 | 0.15 | 0.15 |
| HEATR2 | HEAT repeat-containing protein 2 |  |  | 0.07 | 0.04 |
| HNRNPH3 | Heterogeneous nuclear ribonucleoprotein H3 |  | 0.09 |  | 0.09 |
| EIF4H | Eukaryotic translation initiation factor 4H |  | 0.12 |  | 0.26 |
| LRRC40 | Leucine-rich repeat-containing protein 40 |  | 0.05 | 0.15 | 0.05 |
| LYN | Tyrosine-protein kinase Lyn |  |  | 0.06 | 0.06 |
| MCM2 | DNA replication licensing factor MCM2 | 0.03 |  | 0.07 | 0.07 |
| MCTS1 | Malignant T-cell-amplified sequence 1 | 0.16 | 0.16 | 0.35 | 0.35 |
| MST4 | Serine/threonine-protein kinase MST4 |  | 0.07 | 0.07 | 0.07 |
| NAMPT | Nicotinamide phosphoribosyltransferase | 0.06 | 0.12 | 0.12 | 0.12 |
| NDUFS1 | NADH-ubiquinone oxidoreductase 75 kDa subunit, mitochondrial |  | 0.04 |  | 0.04 |
| NXF1 | Nuclear RNA export factor 1 |  |  | 0.05 | 0.05 |
| DBT | Lipoamide acyltransferase component of branched-chain alpha-keto acid dehydrogenase complex, mitochondrial |  |  | 0.06 | 0.06 |
| PABPC1 | Polyadenylate-binding protein 1 | 0.05 | 0.1 | 0.1 | 0.15 |
| PFKL | ATP-dependent 6-phosphofructokinase, liver type | 0.04 | 0.08 | 0.12 | 0.04 |
| PRPF8 | Pre-mRNA-processing-splicing factor 8 | 0.02 | 0.04 | 0.01 | 0.09 |
| PSMD10 | 26S proteasome non-ATPase regulatory subunit 10 |  |  | 0.14 | 0.07 |
| PSMG1 | Proteasome assembly chaperone 1 |  |  | 0.1 | 0.1 |
| PAICS | Multifunctional protein ADE2 |  | 0.23 |  | 0.15 |
| ADSL | Adenylosuccinate lyase | 0.06 | 0.12 | 0.06 | 0.12 |
| RAB21 | Ras-related protein Rab-21 |  | 0.14 | 0.14 | 0.14 |
| RBM39 | RNA-binding protein 39 |  |  | 0.11 | 0.06 |
| RPL35 | 60S ribosomal protein L35 |  | 0.52 | 0.52 | 0.52 |
| RPL35A | 60S ribosomal protein L35a |  | 0.27 |  | 0.27 |
| TRIM21 | E3 ubiquitin-protein ligase TRIM21 |  |  | 0.06 | 0.06 |
| ROA0 | Heterogeneous nuclear ribonucleoprotein A0 |  | 0.11 | 0.23 | 0.23 |
| RRAGA | Ras-related GTP-binding protein A |  | 0.19 |  | 0.19 |
| RPS12 | 40S ribosomal protein S12 | 0.52 | 1.32 | 1.86 | 1.32 |
| RPS19 | 40S ribosomal protein S19 | 0.21 | 0.77 | 0.77 | 0.47 |
| RPS3 | 40S ribosomal protein S3 | 1.57 | 2.25 | 4.22 | 3.12 |
| RPS5 | 40S ribosomal protein S5 |  | 0.31 | 0.31 | 0.15 |
| SNRPE | Small nuclear ribonucleoprotein E |  |  | 0.32 | 0.32 |
| SDHB | Succinate dehydrogenase [ubiquinone] iron-sulfur subunit, mitochondrial |  | 0.22 | 0.11 | 0.11 |
| SIPA1 | Signal-induced proliferation-associated protein 1 |  |  | 0.09 | 0.03 |
| SKIV2L2 | Superkiller viralicidic activity 2-like 2 |  | 0.03 |  | 0.03 |
| SMC4 | Structural maintenance of chromosomes protein 4 |  |  | 0.02 | 0.02 |
| SMCHD1 | Structural maintenance of chromosomes flexible hinge domain-containing protein 1 |  |  | 0.01 | 0.01 |
| SRP14 | Signal recognition particle 14 kDa protein |  |  | 0.23 | 0.23 |
| SRP68 | Signal recognition particle subunit SRP68 | 0.05 | 0.05 | 0.1 | 0.15 |
| STOML2 | Stomatin-like protein 2, mitochondrial | 0.09 | 0.09 | 0.28 | 0.18 |
| GARS | Glycine--tRNA ligase | 0.08 | 0.12 | 0.21 | 0.21 |
| NARS | Asparagine--tRNA ligase, cytoplasmic |  | 0.05 | 0.05 | 0.11 |
| CCT6B | T-complex protein 1 subunit zeta-2 |  | 0.18 | 0.18 | 0.18 |
| TRAP1 | Heat shock protein 75 kDa, mitochondrial | 0.04 | 0.13 | 0.04 | 0.17 |
| TXNDC17 | Thioredoxin domain-containing protein 17 |  |  | 0.24 | 0.24 |
| EFTUD2 | 116 kDa U5 small nuclear ribonucleoprotein component | 0.03 | 0.06 | 0.03 | 0.09 |
| UQCRFS1P1 | Putative cytochrome b-c1 complex subunit Rieske-like protein 1 | 0.11 | 0.36 | 0.23 | 0.23 |
| RPS6 | 40S ribosomal protein S6 | 0.39 | 0.93 | 0.73 | 0.93 |
| ACAT2 | Acetyl-CoA acetyltransferase, cytosolic |  | 0.08 |  | 0.08 |
| PGD | 6-phosphogluconate dehydrogenase, decarboxylating | 0.13 | 0.2 | 0.27 | 0.27 |
| AGPS | Alkyldihydroxyacetonephosphate synthase, peroxisomal |  | 0.05 |  | 0.09 |
| ADD1 | Alpha-adducin |  | 0.04 | 0.04 | 0.04 |
| AIFM1 | Apoptosis-inducing factor 1, mitochondrial | 0.05 | 0.1 | 0.05 | 0.1 |
| Downregulated proteins after SIM deletion | | | | | |
| UBP7 | Ubiquitin carboxyl-terminal hydrolase 7 | 0.35 | 0.16 | 0.05 | 0.11 |
| C1QBP | Complement component 1 Q subcomponent-binding protein, mitochondrial | 3.54 | 1.24 | 1.24 | 1.48 |
| PI4KB | Phosphatidylinositol 4-kinase beta | 0.15 | 0.04 | 0.04 | 0.04 |
| HSPA8 | Heat shock cognate 71 kDa protein | 0.72 | 0.5 | 0.25 | 0.5 |
| PFAS | Phosphoribosylformylglycinamidine synthase | 0.07 |  | 0.02 | 0.05 |
| KRT9 | Keratin, type I cytoskeletal 9 | 2.11 | 1.06 | 0.86 | 1.28 |
| RPL10A | 60S ribosomal protein L10a | 0.88 | 0.46 | 0.88 | 0.29 |
| EIF3B | Eukaryotic translation initiation factor 3 subunit B | 0.11 |  | 0.04 | 0.04 |
| RRP12 | RRP12-like protein | 0.05 | 0.02 |  | 0.02 |
| RPL27 | 60S ribosomal protein L27 | 1.18 | 1.18 | 0.48 | 0.48 |
| PDCD6IP | Programmed cell death 6-interacting protein | 0.07 | 0.03 | 0.03 | 0.03 |
| SRRT | Serrate RNA effector molecule homolog | 0.07 | 0.07 | 0.07 | 0.03 |
| RPL17 | 60S ribosomal protein L17 | 0.79 | 0.34 | 0.34 | 0.34 |
| RPS26 | 40S ribosomal protein S26 | 0.6 | 0.26 | 0.26 | 1.02 |
| RPS8 | 40S ribosomal protein S8 OS=Homo sapiens GN=RPS8 PE=1 SV=2 | 0.68 | 0.91 | 0.3 | 0.3 |
| RPL27A | 60S ribosomal protein L27a | 0.45 |  | 0.2 | 0.45 |
| ABCD3 | ATP-binding cassette sub-family D member 3 | 0.09 | 0.04 | 0.04 | 0.04 |
| NUP85 | Nuclear pore complex protein Nup85 | 0.09 | 0.04 |  | 0.09 |
| RPL28 | 60S ribosomal protein L28 | 0.48 | 0.22 | 0.48 | 1.18 |
| ALYREF | THO complex subunit 4 | 0.26 | 0.12 | 0.12 | 0.12 |
| RPL13A | 60S ribosomal protein L13a | 0.3 |  | 0.14 | 0.14 |
| WDR77 | Methylosome protein 50 | 0.19 | 0.09 | 0.09 | 0.09 |
| FBL | rRNA 2~-O-methyltransferase fibrillarin | 0.21 | 0.1 | 0.1 | 0.32 |
| GSTM3 | Glutathione S-transferase Mu 3 | 0.27 | 0.13 | 0.13 | 0.27 |
| RAB35 | Ras-related protein Rab-35 | 0.31 | 0.15 | 0.15 |  |
| SLC25A1 | Tricarboxylate transport protein, mitochondrial | 0.2 | 0.2 | 0.2 | 0.1 |
| ACTR1B | Beta-centractin | 0.16 |  | 0.16 |  |
| PRIM2 | DNA primase large subunit | 0.12 | 0.12 |  | 0.06 |
| RSL1D1 | Ribosomal L1 domain-containing protein 1 | 0.12 | 0.06 | 0.12 | 0.06 |
| PSMD1 | 26S proteasome non-ATPase regulatory subunit 1 | 0.06 | 0.03 | 0.03 | 0.03 |
| ITPR3 | Inositol 1,4,5-trisphosphate receptor type 3 | 0.02 |  |  | 0.01 |
| PRPF8 | Pre-mRNA-processing-splicing factor 8 | 0.02 | 0.04 | 0.01 | 0.09 |
| ACTB | Actin, cytoplasmic 1 | 25.51 |  | 88.75 |  |
| DCD | Dermcidin | 0.7 |  |  |  |
| CHCHD2 | Coiled-coil-helix-coiled-coil-helix domain-containing protein 2, mitochondrial | 0.48 | 0.22 | 0.09 | 0.18 |
| CSNK2A1 | Casein kinase II subunit alpha | 0.33 |  |  |  |
| RAB13 | Ras-related protein Rab-13 | 0.32 |  | 0.32 |  |
| RAB1A | Ras-related protein Rab-1A | 0.32 |  |  |  |
| PPP1CA | Serine/threonine-protein phosphatase PP1-alpha catalytic subunit | 0.29 |  |  | 0.29 |
| RAB15 | Ras-related protein Rab-15 | 0.29 |  |  |  |
| YWHAH | 14-3-3 protein eta | 0.25 |  | 0.25 |  |
| EIF2S3 | Eukaryotic translation initiation factor 2 subunit 3 | 0.21 | 0.37 | 0.37 |  |
| TOMM20 | Mitochondrial import receptor subunit TOM20 homolog | 0.21 |  |  | 0.21 |
| HS90A5 | Putative heat shock protein HSP 90-alpha A5 | 0.18 |  | 0.18 |  |
| GNAT3 | Guanine nucleotide-binding protein G(t) subunit alpha-3 | 0.17 |  | 0.17 | 0.05 |
| NAA50 | N-alpha-acetyltransferase 50 | 0.17 |  |  |  |
| CBX3 | Chromobox protein homolog 3 | 0.16 |  |  |  |
| PDHA1 | Pyruvate dehydrogenase E1 component subunit alpha, somatic form, mitochondrial | 0.16 | 0.08 |  | 0.08 |
| PGRMC1 | Membrane-associated progesterone receptor component 1 | 0.16 |  | 0.16 |  |
| PRDX1 | Peroxiredoxin-1 | 0.15 |  |  |  |
| TIMM23 | Mitochondrial import inner membrane translocase subunit Tim23 | 0.15 | 0.15 |  |  |
| RAB6A | Ras-related protein Rab-6A | 0.14 |  |  |  |
| RAB7A | Ras-related protein Rab-7a | 0.14 |  |  |  |
| RL10L | 60S ribosomal protein L10-like | 0.14 |  |  |  |
| RARS | Arginine--tRNA ligase, cytoplasmic | 0.14 | 0.04 | 0.04 | 0.04 |
| ICLN_HUMAN | Methylosome subunit pICln OS=Homo sapiens GN=CLNS1A PE=1 SV=1 | 0.13 |  | 0.13 |  |
| PRDX6 | Peroxiredoxin-6 | 0.13 |  |  |  |
| RB33B | Ras-related protein Rab-33B | 0.13 |  |  |  |
| RU2B | U2 small nuclear ribonucleoprotein B~ | 0.13 |  |  |  |
| SAMP | Serum amyloid P-component | 0.13 |  |  |  |
| LN28B_ | Protein lin-28 homolog B | 0.12 |  |  |  |
| PNO1 | RNA-binding protein PNO1 | 0.12 |  |  |  |
| PRDX3 | Thioredoxin-dependent peroxide reductase, mitochondrial | 0.12 |  |  |  |
| SPR | Sepiapterin reductase | 0.12 |  |  |  |
| PHB | Prohibitin | 0.11 |  |  | 0.24 |
| VDAC2 | Voltage-dependent anion-selective channel protein 2 | 0.11 | 0.11 |  |  |
| AIMP1 | Aminoacyl tRNA synthase complex-interacting multifunctional protein 1 | 0.1 |  |  |  |
| NB5R3 | NADH-cytochrome b5 reductase 3 | 0.1 |  |  |  |
| SYK | Lysine--tRNA ligase | 0.1 | 0.15 |  | 0.15 |
| TACO1 | Translational activator of cytochrome c oxidase 1 | 0.1 |  |  |  |
| GBB1 | Guanine nucleotide-binding protein G(I)/G(S)/G(T) subunit beta-1 | 0.09 | 0.18 |  |  |
| GRHPR | Glyoxylate reductase/hydroxypyruvate reductase | 0.09 | 0.09 |  | 0.09 |
| NUP37 | Nucleoporin Nup37 | 0.09 | 0.19 |  | 0.09 |
| PRKDC | DNA-dependent protein kinase catalytic subunit | 0.09 | 0.02 | 0.03 | 0.03 |
| SF3B1 | Splicing factor 3B subunit 1 | 0.09 |  |  | 0.02 |
| STRAP | Serine-threonine kinase receptor-associated protein | 0.09 |  |  | 0.09 |
| ADHX | Alcohol dehydrogenase class-3 | 0.08 |  | 0.08 |  |
| CSK22 | Casein kinase II subunit alpha | 0.08 |  |  |  |
| DCUP | Uroporphyrinogen decarboxylase | 0.08 | 0.08 |  | 0.08 |
| DDX50 | ATP-dependent RNA helicase DDX50 | 0.08 |  |  |  |
| TIM50 | Mitochondrial import inner membrane translocase subunit TIM50 | 0.08 | 0.08 |  |  |
| ERR1 | Steroid hormone receptor ERR1 | 0.07 |  |  | 0.07 |
| HAT1 | Histone acetyltransferase type B catalytic subunit | 0.07 | 0.07 |  | 0.14 |
| HPT | Haptoglobin | 0.07 |  |  |  |
| SND1 | Staphylococcal nuclease domain-containing protein 1 | 0.07 | 0.07 | 0.03 |  |
| WDR18 | WD repeat-containing protein 18 | 0.07 |  |  | 0.07 |
| AAAS | Aladin | 0.06 | 0.06 |  | 0.06 |
| DNJA3 | DnaJ homolog subfamily A member 3, mitochondrial | 0.06 | 0.06 |  | 0.06 |
| FSCN1 | Fascin | 0.06 | 0.06 | 0.06 |  |
| IMA4 | Importin subunit alpha-4 | 0.06 |  |  | 0.06 |
| IMDH1 | Inosine-5~-monophosphate dehydrogenase 1 | 0.06 |  |  |  |
| MOT1_HUMAN | Monocarboxylate transporter 1 OS=Homo sapiens GN=SLC16A1 PE=1 SV=3 | 0.06 | 0.06 |  |  |
| TBCE_HUMAN | Tubulin-specific chaperone E OS=Homo sapiens GN=TBCE PE=1 SV=1 | 0.06 | 0.06 |  |  |
| ALB | Serum albumin | 0.05 |  |  |  |
| PRMT5 | Protein arginine N-methyltransferase 5 | 0.05 | 0.05 |  | 0.05 |
| CPNE2 | Copine-2 | 0.05 |  | 0.05 |  |
| PGBD4 | PiggyBac transposable element-derived protein 4 | 0.05 |  |  |  |
| STXBP3 | Syntaxin-binding protein 3 | 0.05 |  | 0.05 | 0.1 |
| ACSL3 | Long-chain-fatty-acid--CoA ligase 3 | 0.04 | 0.04 |  |  |
| CUL4A | Cullin-4A | 0.04 |  | 0.12 | 0.08 |
| GLS | Glutaminase kidney isoform, mitochondrial | 0.04 |  | 0.04 |  |
| RECQ1 | ATP-dependent DNA helicase Q1 | 0.04 |  |  |  |
| SRP72 | Signal recognition particle subunit SRP72 | 0.04 |  | 0.04 |  |
| ZNF81 | Zinc finger protein 81 | 0.04 |  |  |  |
| EPB41 | Protein 4.1 | 0.03 |  |  |  |
| C4A | Complement C4-A =2 | 0.03 |  |  |  |
| FLNC | Filamin-C | 0.03 | 0.02 |  | 0.02 |
| GANAB | Neutral alpha-glucosidase AB | 0.03 |  |  |  |
| IPO5 | Importin-5 | 0.03 |  |  |  |
| MAML2 | Mastermind-like protein 2 | 0.03 |  |  |  |
| NU107 | Nuclear pore complex protein Nup107 | 0.03 |  |  |  |
| FTSJ3 | pre-rRNA processing protein FTSJ3 | 0.03 | 0.03 |  |  |
| ABCC9 | ATP-binding cassette sub-family C member 9 | 0.02 |  |  |  |
| CLUH | Clustered mitochondria protein homolog | 0.02 | 0.04 |  | 0.09 |
| MAP3K1 | Mitogen-activated protein kinase kinase kinase 1 | 0.02 |  |  |  |
| MYO1B | Unconventional myosin-Ib | 0.02 |  |  | 0.08 |
| PALLD | Palladin | 0.02 |  |  |  |
| SPTBN4 | Spectrin beta chain, non-erythrocytic 4 | 0.02 | 0.03 | 0.02 |  |
