# Supplementary figures and images for: STUB1 is targeted by the SUMO-interacting motif of EBNA1 to maintain Epstein-Barr Virus latency
Source: PLoS Pathog. 2020 Mar 16;16(3):e1008447. doi: 10.1371/journal.ppat.1008447 (PMC7105294; doi:10.1371/journal.ppat.1008447)

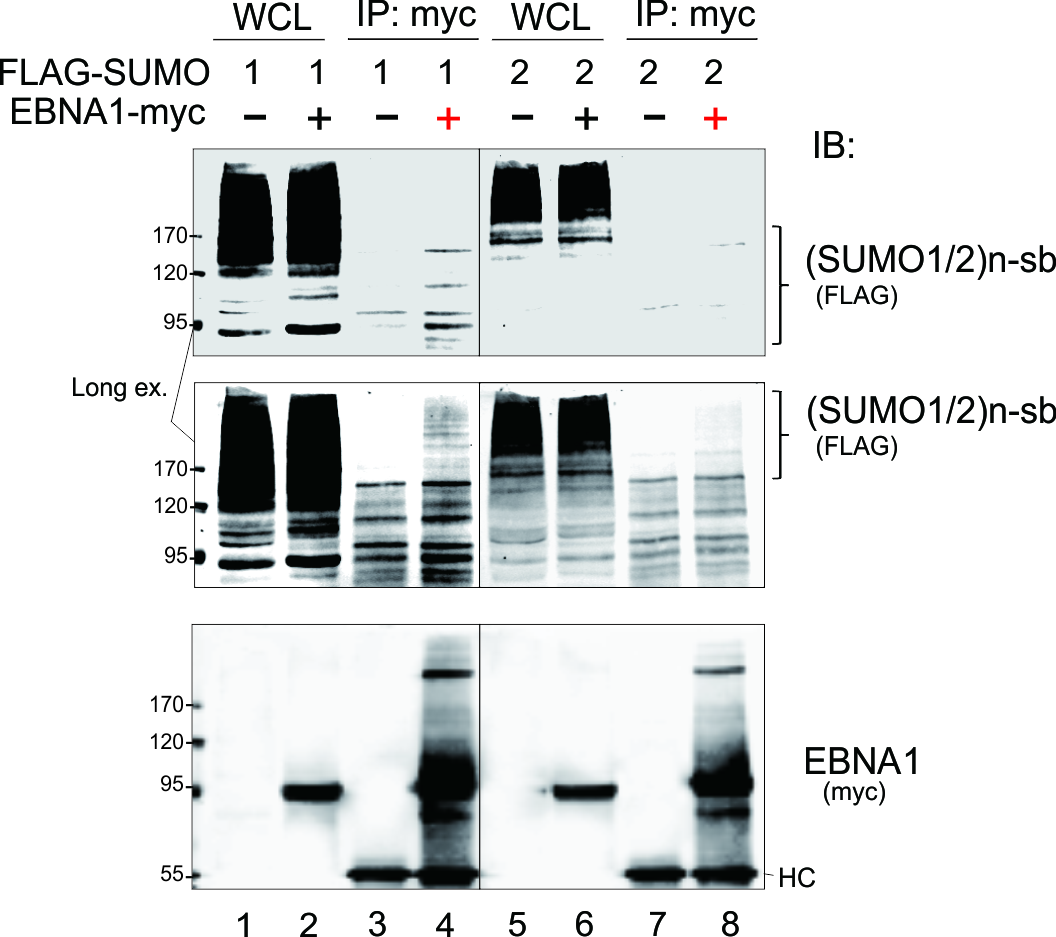

Supplement: S1 Fig — HEK293 cells were co-transfected with expression plasmids as indicated in the figure. At 48 post-transfection, whole cell lysates were subjected to immunoprecipitated (IP) and immunoblotting (IB) as indicated. The position (>170 kDa) of EBNA1-interacting SUMO1 or SUMO2 modified substrates [(SUMO1/2)n-sb] is highlighted. (TIF) [file ppat.1008447.s005.tif]

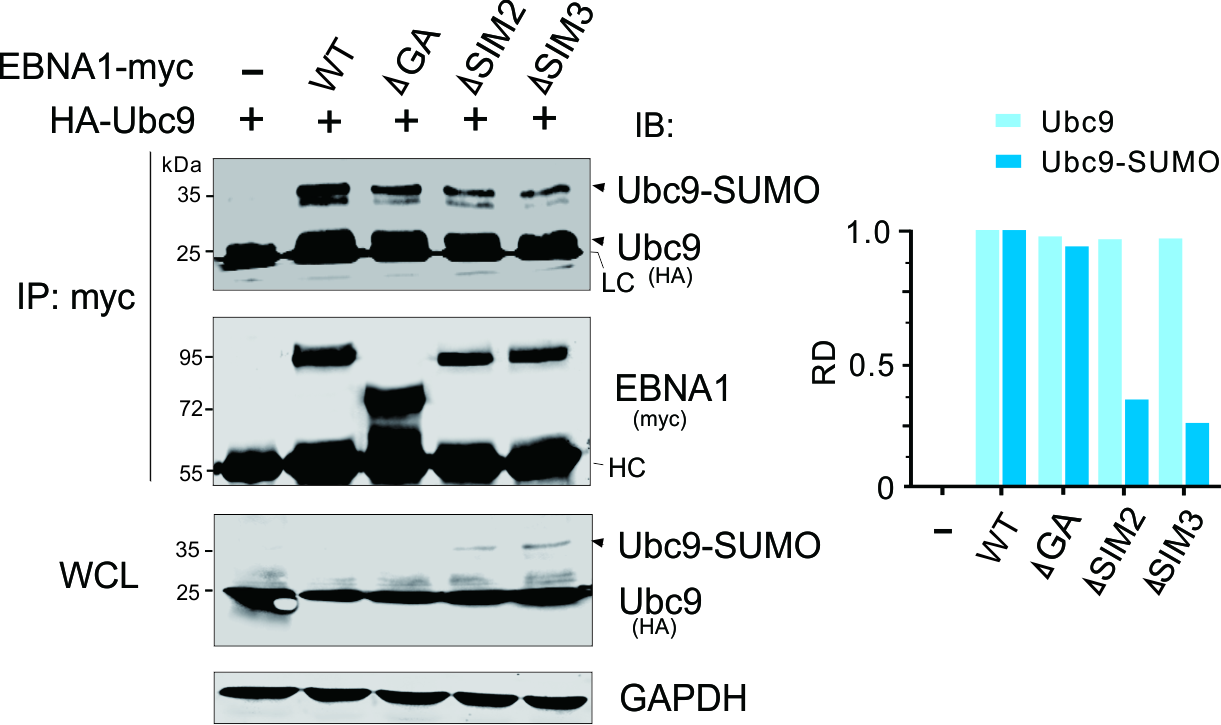

Supplement: S2 Fig — HEK293T cells were co-transfected with expression plasmids as indicated. Whole cell lysates (WCL) were harvested at 48 h post-transfection, and subjected to co-IP and IB as indicated. The relative density (RD) of EBNA1-binding Ubc9 and Ubc9-SUMO is quantified and shown on the right panel. (TIF) [file ppat.1008447.s006.tif]

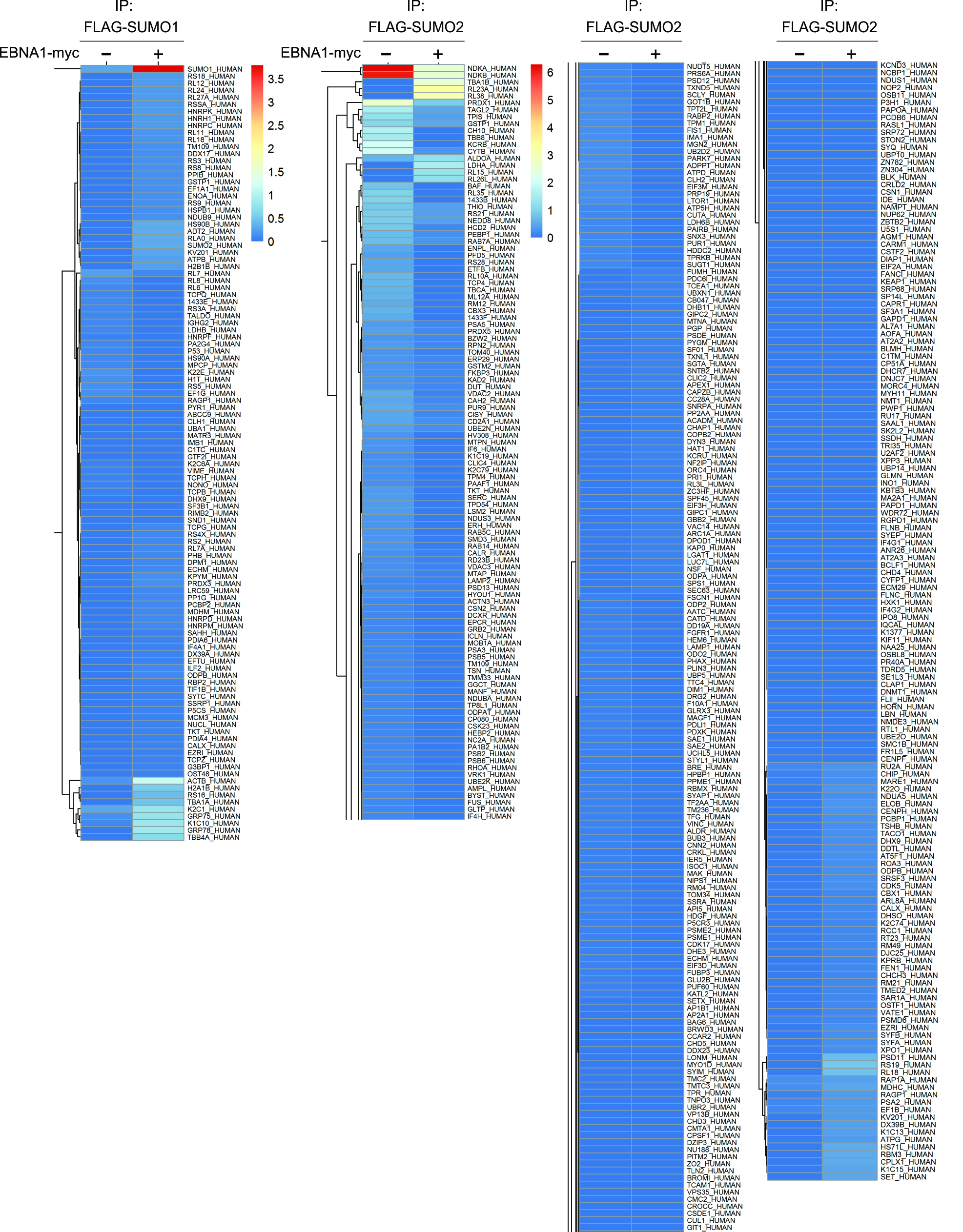

Supplement: S3 Fig — Related to Fig 4A. (TIF) [file ppat.1008447.s007.tif]

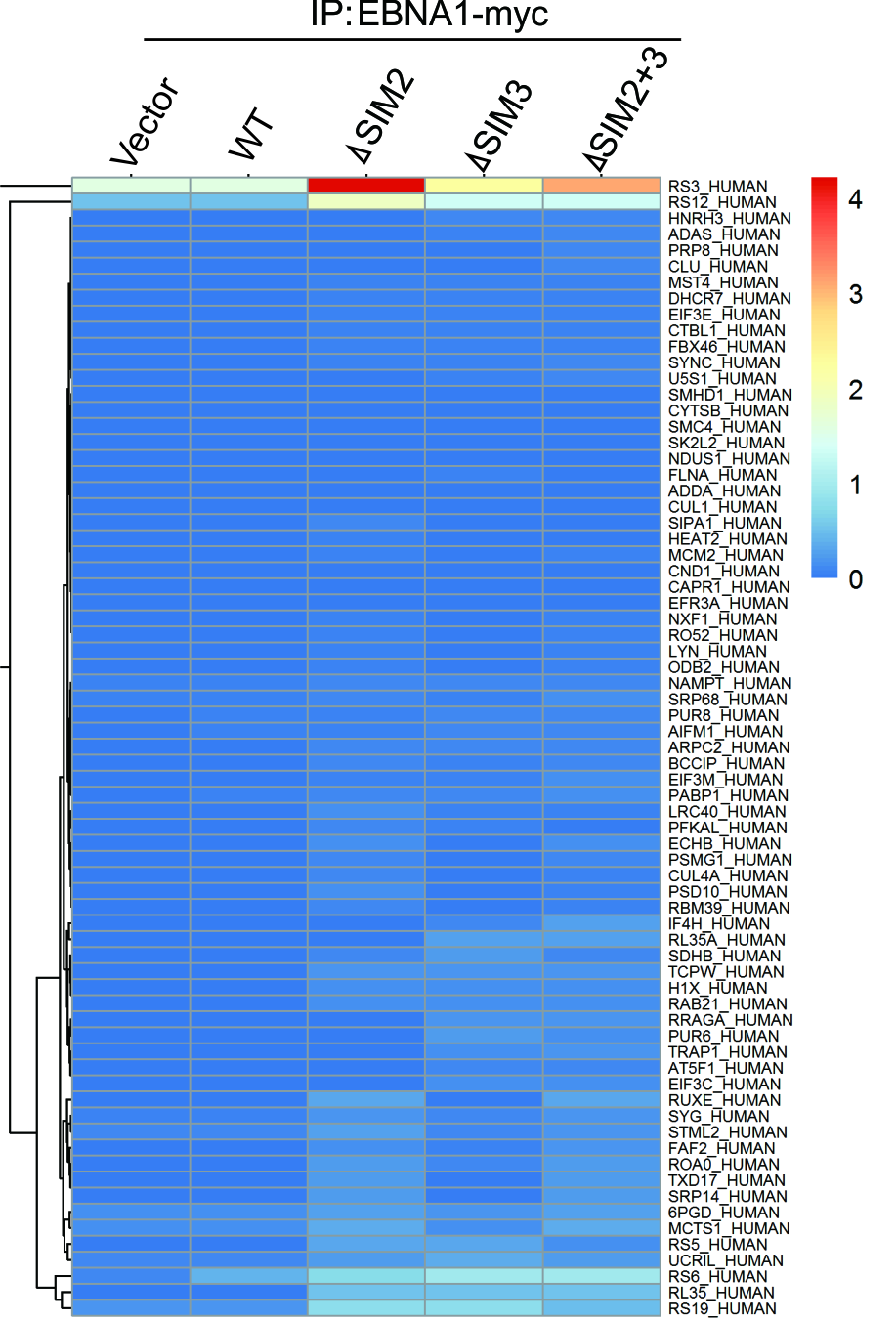

Supplement: S4 Fig — Related to Fig 6A. (TIF) [file ppat.1008447.s008.tif]

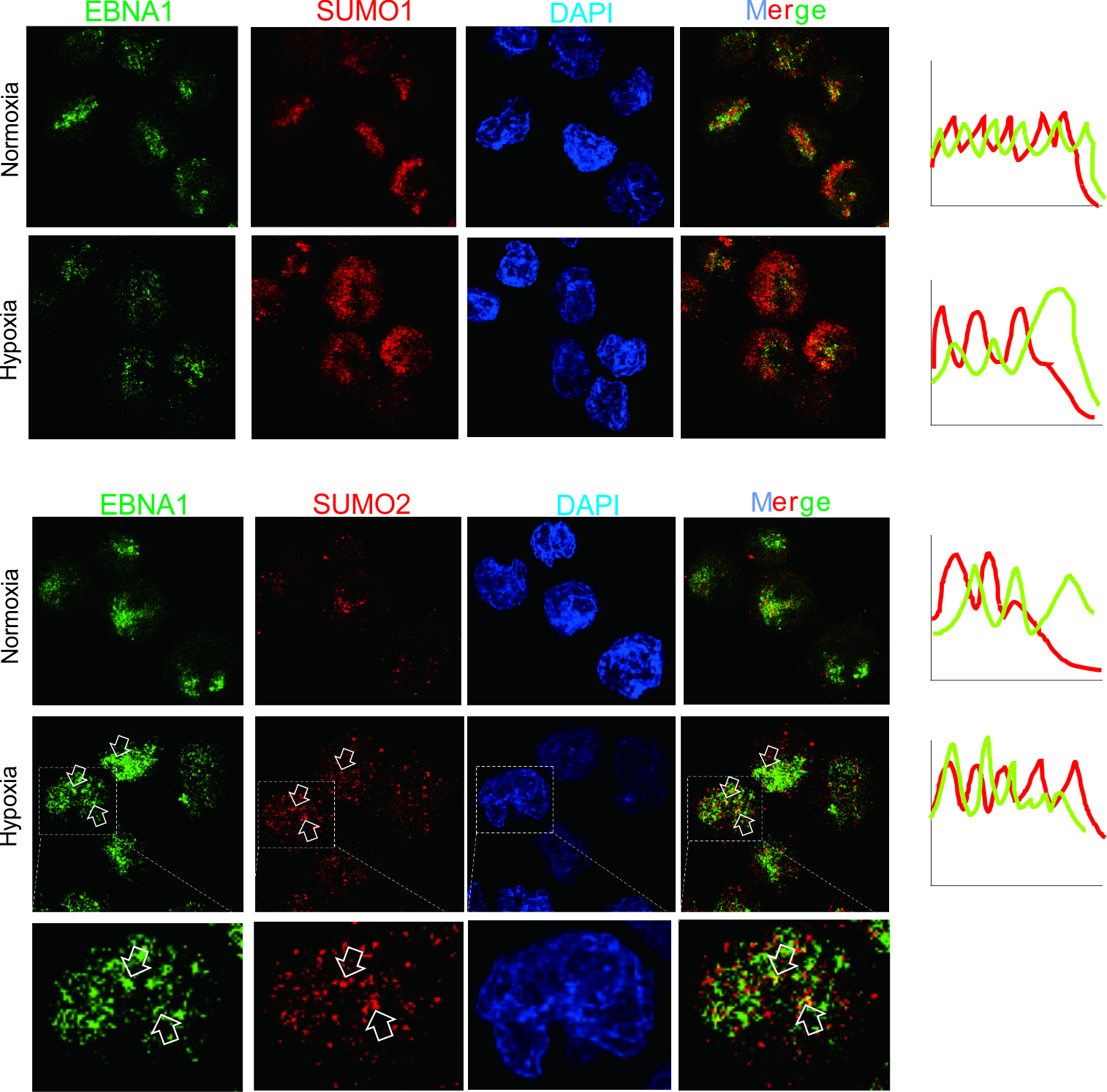

Supplement: S5 Fig — LCL1 cells were subjected to hypoxia (0.2% oxygen) treatment for overnight. Endogenous EBNA1, SUMO1, and SUMO2 were individually stained by EBNA1 (green) and SUMO1/2 (red) antibodies. The profile of EBNA1 and SUMO1/2 immunofluorescence were quantified and shown on the right panels. SUMO2 co-localization with EBNA1 was highlighted by the arrows and enlarged at the bottom panels. (TIF) [file ppat.1008447.s009.tif]

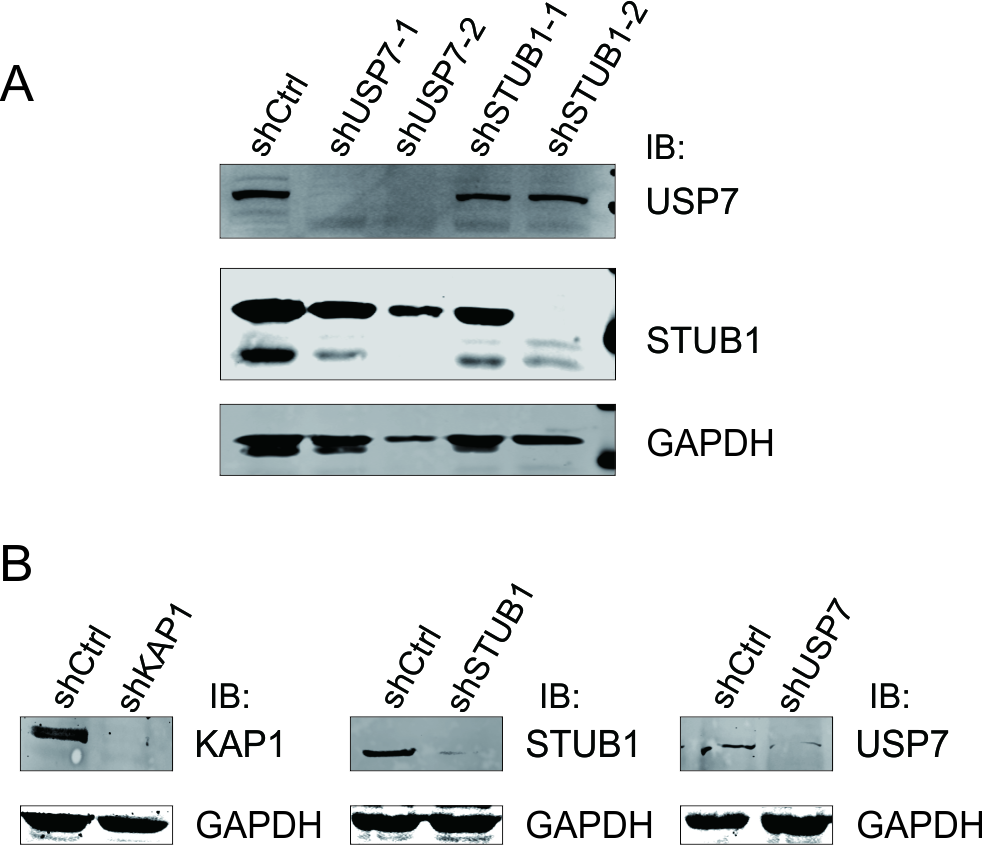

Supplement: S6 Fig — Whole cell lysate of 293T (A) or LCL (B) cells with KAP1, STUB1 or USP7 knockdown were individually subjected to immunoblotting (IB) with the indicated antibodies. The luciferase knockdown (shCtrl) was used as control. (TIF) [file ppat.1008447.s010.tif]
